# Modified Snake α-Neurotoxin Averts β-Amyloid Binding to α7 Nicotinic Acetylcholine Receptor and Reverses Cognitive Deficits in Alzheimer’s Disease Mice

**DOI:** 10.1007/s12035-020-02270-0

**Published:** 2021-01-08

**Authors:** Gennadiy Fonar, Baruh Polis, Dev Sharan Sams, Almog Levi, Assaf Malka, Natalia Bal, Alexander Maltsev, Evan Elliott, Abraham O. Samson

**Affiliations:** 1 Drug Discovery Laboratory, The Azrieli Faculty of Medicine, Bar-Ilan University, 1311502 Safed, Israel; 2 Laboratory of Molecular and Behavioral Neuroscience, The Azrieli Faculty of Medicine, Bar-Ilan University, 1311502 Safed, Israel; 3 Institute of Higher Nervous Activity and Neurophysiology, Russian Academy of Sciences, Moscow, Russia

**Keywords:** Alzheimer’s disease, Snake α-neurotoxin, Amyloid-beta, Cholinergic hypothesis, Nicotinic acetylcholine receptor

## Abstract

Alzheimer’s disease (AD) is the most common cause of senile dementia and one of the greatest medical, social, and economic challenges. According to a dominant theory, amyloid-β (Aβ) peptide is a key AD pathogenic factor. Aβ-soluble species interfere with synaptic functions, aggregate gradually, form plaques, and trigger neurodegeneration. The AD-associated pathology affects numerous systems, though the substantial loss of cholinergic neurons and α7 nicotinic receptors (α7AChR) is critical for the gradual cognitive decline. Aβ binds to α7AChR under various experimental settings; nevertheless, the functional significance of this interaction is ambiguous. Whereas the capability of low Aβ concentrations to activate α7AChR is functionally beneficial, extensive brain exposure to high Aβ concentrations diminishes α7AChR activity, contributes to the cholinergic deficits that characterize AD. Aβ and snake α-neurotoxins competitively bind to α7AChR. Accordingly, we designed a chemically modified α-cobratoxin (mToxin) to inhibit the interaction between Aβ and α7AChR. Subsequently, we examined mToxin in a set of original in silico, in vitro*,* ex vivo experiments, and in a murine AD model. We report that mToxin reversibly inhibits α7AChR, though it attenuates Aβ-induced synaptic transmission abnormalities, and upregulates pathways supporting long-term potentiation and reducing apoptosis. Remarkably, mToxin demonstrates no toxicity in brain slices and mice. Moreover, its chronic intracerebroventricular administration improves memory in AD-model animals. Our results point to unique mToxin neuroprotective properties, which might be tailored for the treatment of AD. Our methodology bridges the gaps in understanding Aβ-α7AChR interaction and represents a promising direction for further investigations and clinical development.

**Supplementary Information:**

The online version contains supplementary material available at 10.1007/s12035-020-02270-0.

## Introduction

Alzheimer’s disease (AD) is a severe neurodegenerative disorder characterized by the gradual accumulation of misfolded proteins and their fragments in the brain, progressive neuronal loss, and neuroinflammation, followed by the steady and inevitable decline in memory and other cognitive functions [1]. At present, AD is an irredeemable pathology presenting one of the most significant medical, social, and economic challenges. Despite a century-long laborious investigation, no complete scientific consensus regarding the causes of AD has been achieved. Several hypotheses have been proposed; however, neither of them explains the complexity of AD pathogenesis or elucidates its etiology.

The cholinergic hypothesis of AD, introduced some 40 years ago, suggests that a dysfunction of cholinergic neurons in the brain contributes to the cognitive decline. From the time of its initial articulation [2], this theory stimulated substantial scientific interest and generated widespread continuous debate and controversy [3]. Mounting clinical and experimental evidence has proven the role of cholinergic dysfunction in the development of AD-associated cognitive decline, and thus offered a promising treatment approach. In fact, the most efficient to date treatment strategies and drug development approaches have been predicated upon the theoretical basis of the cholinergic hypothesis. Initial preclinical studies with cholinomimetic agents, and physostigmine in particular, in monkeys [4] have been followed by numerous clinical trials, which eventually resulted in the approval by the Food and Drug Administration (FDA) of Tacrine as the very first drug targeting memory and thinking problems associated with AD. Currently, there are three FDA-approved and commonly prescribed cholinesterase inhibitors that relieve the symptoms of AD-associated dementia [5]. Inhibitors of acetylcholinesterase decrease the rate of acetylcholine (ACh) breakdown and improve cholinergic neurotransmission, even though their efficacy is noticeably limited [6].

Later discoveries and identification of a cerebrovascular amyloid protein, also known as amyloid β (Aβ), which constitutes the principal component of senile plaques [7], led to the formulation of the amyloid cascade hypothesis that has become the dominant model of AD pathogenesis for more than two decades [1]. This theory proclaims that Aβ is a prime suspect in triggering AD-associated nerve cell damage [8]. This thesis has been guiding the research and development of novel potential medicines; however, efforts to find efficient disease-modifying therapy have been of no avail. A cornucopia of potential agents has been trialed, though, with no irrefutable results. The enormous complexity of AD pathogenesis and impossibility to detect the precise etiological factor in many cases of the late-onset AD led a clear paradigm shift and formulation of alternative hypotheses, which treat AD as a continuous spectrum of diseases [9] and as a heterogenous syndrome [10]. This concept paves the way for cardinally novel and personalized treatment strategies, which include past achievements together with the latest insights. Here we propose an original methodology, which is based upon the two canonical hypotheses of AD. We target cholinergic synapses with an agent that interferes with the interaction between Aβ and neuronal α7-acetylcholine receptor (α7AChR). Of note, these receptors are highly expressed in the hippocampus, cortex, and limbic brain areas, and are principally involved in cognitive functions, sensory information processing, attention, and reward [11]. A significant reduction in α7AChR levels is reported in AD patients [12], which strongly supports the cholinergic hypothesis.

Nicotinic acetylcholine receptors are a family of ligand-gated ion channels that respond to the neurotransmitter ACh. To date, 17 different subunits (α1–10, β1–4, δ, ε, and γ) have been identified in humans, which can combine and generate many subtypes of homo- and hetero-pentameric AChR with different physiology, pharmacology, and anatomical distributions [13]. Two major subtypes exist in the brain, namely those comprised of α7 and those consisting of α4β2. Of note, a list of AChR binding molecules, such as nicotine, cytisine, and epibatidine, demonstrate agonistic properties; however, several proteins act antagonistically, such as d-tubocurarine, lophotoxins, and snake α-neurotoxins [14].

α7AChR responds to ACh binding by opening a cations permeable intrinsic channel, which leads to membrane depolarization [15]. Also, α7AChR acts as a metabotropic receptor that induces several pathways associated with neuroprotection and synaptic plasticity [16]. The metabotropic effects include MAPK/ERK, PI3K/AKT signaling pathways activation, and mobilization of intracellular calcium reserves [17].

Aβ is derived from the amyloid precursor protein (APP) through sequential cleavage by proteolytic enzymes such as β-secretase and γ-secretase [18]. Soluble Aβ oligomeric species disrupt synaptic function and induce neuronal loss [19]. Several membrane proteins are capable of binding Aβ and, therefore, mediate Aβ-associated toxicity [20]. Remarkably, Aβ binds to neuronal α7AChR with high affinity and blocks cholinergic neurotransmission [21]. This process results in blockage of α7AChR channels [22], receptor internalization, gradual intracellular accumulation of Aβ-α7AChR complexes, which is accompanied by severe impairment of cholinergic neurons function [23], and, eventually, neuronal cell death. Recently, robust data elucidated the intricate interaction between Aβ and α7AChR [24]. It has been shown that Aβ dualistically affects α7AChR channel function and acts as an agonist and a negative modulator. At high concentrations, Aβ inhibits α7AChR and blocks the channel by acting at an allosteric site. However, at low concentrations, Aβ acts as a low-efficacy agonist, and prolonged exposure to Aβ species reduces the α7AChR activity, leads to the characteristic cholinergic signaling deficit, and contributes to further development of clinical symptoms [24]. It is worth noting that the dual-mode action of Aβ on α7AChR activity is not unique. Several compounds have been described in the literature that act in opposite ways at different sites of α7AChR as low-efficacy agonists or as channel blockers in a concentration dependent manner [25].

Accumulating data suggest an additional lower affinity Aβ binding site, which partially overlaps with the agonist binding site at α7AChR. According to this model, prolonged exposure to high concentrations of Aβ induces a conformational change in the receptor, which makes subsequent activation by natural agonists much less efficient [21, 26].

Snake venom α-neurotoxins bind to α7AchR and competitively inhibit ACh binding, prevent the depolarizing action on postsynaptic membranes, and block neuronal transmission [27]. α-Neurotoxins are divided into two major groups according to their length, namely short α-neurotoxins, comprising approximately 61 residues, and long α-neurotoxins, consisting of approximately 74 amino acids, such as α-cobratoxin (CTX).

Previously, our group has proposed an exclusive binding mechanism of Aβ to neuronal α7AChR predicated upon sequence and structural similarities between Aβ and snake α-neurotoxins [28]. In the current study, we design an original nontoxic modified snake α-neurotoxin, which, in accordance with our in silico modeling, interferes with Aβ binding to α7AChR. We hypothesize that the modified toxin (mToxin) is capable of preventing some of the deleterious effects of Aβ in the brain, which we consequently examine in a series of in vitro, ex vivo, and in vivo experiments. Additionally, we shed light upon the molecular mechanism of the mToxin effects via advanced proteomics and immunohistochemistry assays.

Remarkably, the modified toxin shows no toxicity in vitro and in vivo. It partially inhibits α7AChR for a brief period of several seconds. Moreover, the compound rescues synaptic transmission deficits by blocking Aβ binding to α7AChR and thereby improves memory in AD mice.

Our study significantly updates the current knowledge on the Aβ-α7AChR interaction. Likewise, it demonstrates the functional consequences of this interaction from molecular to cellular and, eventually, cognitive levels. We also discuss the possible implications these relationships might have for prospective AD-modifying therapies and suggest a possible drug design.

## Materials and Methods

### Snake α-Cobratoxin Modification

Purified snake-venom-derived α-cobratoxin (CTX), a long α-neurotoxin, was purchased from Latoxan (Portes-lès-Valence, France). The toxin was chemically modified with phenylglyoxal using a protocol described earlier [29]. Introduced by Kenji Takahashi more than 50 years ago as an efficient arginyl reagent, phenylglyoxal has been applied for the investigation of complex systems and as a reagent for the chemical modification of arginine residues in proteins in the past decade [30]. Phenylglyoxal reacts with the guanido group of arginine residues under mild conditions. The derivative is sufficiently stable and decomposes very slowly [30].

Shortly, the toxin (0.05 mM) was incubated for 1.5 h with an excess of phenylglyoxal (Sigma, St. Louis, Missouri, US) (100 mM) in a 1-ml alkaline solution containing 50 mM sodium pyrophosphate buffer at pH 9. Then, the mToxin was purified by fast protein liquid chromatography (FPLC) using a size exclusion column (GE Sephadex 75) with an ammonium acetate buffer (pH 7). Following purification, the solution was lyophilized and redissolved in 1 ml of water. The mass of the mToxin was verified using matrix-assisted laser desorption/ionization (MALDI) and electrospray ionization (ESI) at the mass spectrometry unit of Bar-Ilan University (Dr. Rachel Persky, Department of Chemistry).

### In Silico Molecular Modeling Analysis

It has been pointed out that Aβ peptide possesses sequential and structural similarities with the functional residues of α-neurotoxins. This observation led to the hypothesis that snake α-neurotoxins competitively inhibit the binding of Aβ to the nicotinic AChR [28]. Consequently, the three-dimensional model of the complex formed by α1AChR and Aβ peptide has been proposed using homology modeling and docking. This model provided a rudimentary glimpse into the interaction between α1AChR and Aβ until their complex is empirically elucidated.

Previous studies have shown that chemical modification of α-neurotoxins with phenylglyoxal dramatically reduces its toxicity [31]. Here, we aim to test the correlation between the toxin’s binding score and its toxicity. We utilized our previously published model (PDB ID ILK1), then virtually modified toxin arginine residues with phenylglyoxal using PyMOL software (Schrödinger) [32], and finally reconstructed their complex with PatchDOCK. In addition, to model the 3D structure of mToxin, we virtually substituted arginine 33 of CTX with phenylglyoxal and reconstructed its complex with α7AChR-like (PDB ID 1YI5) PatchDOCK. PatchDOCK molecular docking algorithm is predicated upon the shape complementarity principles [33], and the standard server parameters were used to predict protein-protein interactions [34]. This computer simulation assessed modified and natural toxins by means of their binding to AChRs. Subsequently, the results were visually examined with PyMOL.

### Manual Patch Clamp Recording

The effects of the modified toxin upon the function of nicotinic acetylcholine receptor in PC12 cell line was tested using manual patch clamp technology by Creative Biogene Inc. (Shirley, NY, USA).

PC12 cells expressing α7AChR were cultured in 1640 medium supplemented with 10% fetal bovine serum and 5% horse serum in a Petri dish. Cells grew in a humidified incubator at 37 °C with 5% CO2. Cells (3×10^3^) were seeded into 24-well plate with a final medium volume of 500 μL and with one coverslip in each well and test after incubation about 18 h (cell density <80%) at 37 °C with 5% CO2.

Nicotinic acetylcholine receptor currents were recorded with a −50 mV pre-test and then recorded under a holding potential at −80 mV. The electrophysiological recordings were obtained under a microscope. The amplifier EPC10 (HEKA Elektronik, Lambrecht, Germany) was used to record the electrophysiological signal. Data were stored and analyzed with Patchmaster (HEKA Elektronik, Lambrecht, Germany) and IGOR Pro (WaveMetrics Inc., Lake Oswego, OR, USA).

Recordings were carried out in extracellular solution composed of 140 mM NaCl, 3.5 mM KCl, 1 mM MgCl_2_, 2 mM CaCl_2_, 10 mM glucose, 10 mM HEPES, 1.25 mM NaH_2_PO_4_ (pH 7.4). Pipette solution: 50 mM CsCl, 10 mM NaCl, 10 mM HEPES, 60 mM CsF, 20 mM EGTA (pH 7.2, pH adjusted with CsOH).

PC12 cells were incubated with a test sample for 30 s. Then five μM PNU-120596 (Sigma), which is a potent and selective positive allosteric modulator for the α7AChR that causes conformational changes in the extracellular ligand-binding domain similar to those caused by ACh, were co-applicated with control or test sample.

In order to study the effect of mToxin on α7AChR AR-R17779 (Sigma) was utilized. This agent is a potent and selective full agonist of these receptors. The test sample was applied gradually. The test or control solutions flowed into a recording chamber mounted on the stage of an inverted microscope via a gravity-fed solution delivery system. Thirty micrometer ACh served as the internal control. All tests were performed at room temperature. The following criteria were used to determine data acceptability: access resistance ≤ 20 MΩ, initial seal resistance ≥ 1 GΩ, initial peak tail current ≥ 400 pA. Within each recording, the current responses to test sample addition were normalized to the ACh vehicle control. Four independent sets of experiments were performed. The chemicals and equipment used are listed in the supplementary Table S1.

### Amyloid-β Aging

A short fragment Aβ_25–35_ (numbers indicate sequence range) self-associates in a similar manner as the full Aβ_1–42_ peptide and quickly converts to an insoluble β-sheet structure. Moreover, Aβ_25–35_ demonstrates a faster fibril formation rate than its longer analog and retains the full-length peptide toxicity [35].

Aβ_25–35_ (Sigma) was aged following a previously published protocol [36]. Briefly, the peptide was dissolved in mQ water to 1 mM stock solution and frozen. Twenty-four hours prior to experiments, the Aβ peptide was dissolved to 50 nM in ACSF (EcoCyte Bioscience, Austin, TX, USA) and incubated at +4 °C for oligomerization.

### Ex Vivo Electrophysiology

The experiments were performed using aged wild type С57BL/6 mice (8–9-month old) (males and females). Animals were quickly decapitated under sevoflurane anesthesia. Brains were submerged in ice-cold dissection solution (concentrations in mM: 124 NaCl, 3 KCl, 1.25 NaH_2_PO_4_, 26 NaHCO_3_, 1.3 CaCl_2_, 7 MgCl_2_, and 10 D-glucose, pH equilibrated with 95% O_2_–5% CO_2_). Transverse slices 350 μm thick were cut from each hippocampus using a vibratome (Leica VT1000S, Germany) and immediately transferred to a recording solution (composition as above, except the CaCl_2_ and MgCl_2_ concentrations, were adjusted to 2.5 and 1.3 mM respectively). Slices were heated to 36 °C in a water bath for 40 min, and then kept at room temperature. Then, they were incubated for 1 h in a control solution (ACSF), 100 nM mToxin solution, 50 nM Aβ_25–35_ peptide or a mixture before transferring to the recording chamber. During the experiments, slices were perfused by a continuously flowing (4 ml/min) recording solution at 32–33 °C.

Electrophysiological recordings were carried out using the SliceMaster system (Scientifica, UK). Field excitatory postsynaptic potentials (fEPSP) were recorded from striatum radiatum in area CA1 using glass microelectrodes (1–2 MΩ) filled with ACSF. Baseline synaptic responses were evoked by paired-pulse stimulation with a 50 ms interval of the Schaffer collaterals at 0.033 Hz with a bipolar electrode (WPI, Sarasota, FL). Test stimulation intensity was adjusted to evoke fEPSP with amplitude 50% of maximal and was kept constant throughout the experiment. Long-term potentiation (LTP) was induced with four 100-Hz trains spaced 5 min apart. The data were recorded and analyzed by Spike2 (Axon Instr., USA) and SigmaPlot (Systat Software Inc., San Jose, CA, USA). For statistical analysis, the data acquired during the last 5 min of the experiment (116–120 min after LTP induction) were used. For baseline responses analysis, fiber volley amplitudes and appropriate fEPSP slopes during test stimulation were evaluated.

Paired-pulse facilitation (PPF) as a form of short-term synaptic plasticity was assessed at interstimulus intervals (ISIs) of 30, 50, 100, 200, 300, and 400 ms in all groups. The paired-pulse ratio was determined as the ratio between the second pulse-evoked and the first one. PPF ratio was calculated as PPF=(S2EPSP/S1EPSP), where S1EPSP and S2EPSP are the slopes of EPSP in response to the first and the second stimuli, respectively.

### Animal Studies

The Institutional Animal Care and Use Committee (IACUC) of Bar Ilan University reviewed and approved the experiments on animals conducted in this study and described below (protocol #32-08–2012).

#### Toxicity Assay

α-Neurotoxins noncovalently bind to nAChR in skeletal muscles, reversibly block the ACh action at the postsynaptic membrane, inhibit ion flow, which eventuates in paralysis and death [37]. CTX is extremely toxic with LD_50_ of about 0.1 mg/kg in rodents [38]. However, the reaction of CTX with phenylglyoxal has been shown to reduce substantially (up to 98.4%) its lethal activity [31].

Considering the CTX paralytic characteristics, we evaluated the toxicity of mToxin in a series of behavioral tests. We doubled the dose that regularly leads to a 50% lethality rate and administered 0.2 mg/kg of the substance intraperitoneally. Eight adult male (18–20 g) 2-month-old C57Bl/6 mice were injected with diluted in PBS toxin solution (0.2 ml per mouse) or with the vehicle. Twenty minutes after the procedure, the animals were observed in the open field and tested in rotarod.

In order to assess general exploratory locomotion, the mice were gently placed into the center of an open field box (50 cm×50 cm) lit with 40 lx of light. The animals were allowed to move freely for 10 min. The total distance moved by each mouse was recorded by a camera (Noldus® Wageningen, NL) connected to the EthoVision® software (Noldus® Wageningen, NL).

We also used a rotarod apparatus ENV-577 M (Med Associates Inc., St. Albans, VT, USA) to measure motor coordination and fatigue resistance. The speed of rotation was gradually increasing from 4 to 40 rpm over 3 min. The mice were placed on the rotating lane of the rotarod, and the timer was started. Animals could remain on the apparatus until they fell off or until 3 min had elapsed. Latency to fall was registered automatically by a photobeam.

#### AD Mice

In order to study the effect of mToxin in vivo in a murine model of AD, triple-transgenic mice (3×Tg) were acquired from Jackson Lab (MMRRC stock #34830) and bred in our animal facility. The mice possess APP Swedish mutation, τ-protein P30IL mutation, and presenilin1 mutation, mimicking the AD-associated pathology, and demonstrate memory deficits from 6 months [39]. Of note, female 7-month-old 3×Tg mice react better to the various treatments than males (Salvatore Oddo, personal communication supported by our results) [40]. Therefore, for the learning and memory experiments, 20 female 7-month-old mice have been divided into two groups. Additionally, 20 age-matched female C57BL/6 mice were used as nontransgenic wild-type (WT) controls in all the experiments.

#### Surgical Procedure

We suspect mToxin does not pass the blood-brain barrier (BBB); thus, to bypass the barrier, the compound was administered directly into the ventricles using osmotic minipumps and cannulae. We applied the same surgical procedure for cannulation, as described previously [36]. Briefly, the mouse skull was drilled in accordance with the coordinates: −0.2 mm caudal, 0.9 mm lateral to bregma. Then, a bent cannula (Alzet) was slowly lowered into the hole and cemented to the skull with Loctite 454 (Alzet). Once in place, the cannula reaches 2.5 mm in the dorsoventral direction. Osmotic Alzet-1004 minipumps for 28 days delivery with a pumping rate of 0.11 μl per hour were filled with 100 μl of mToxin solution (0.05 mM, pH 7.3) or ACSF (Ecocyte Bioscience, USA). The pumps were placed into a subcutaneous pocket and connected to the cannulae via a vinyl catheter tube. The timeline of the experiment, including the treatment and behavioral tests, is presented in Fig. 5a.

#### Behavioral Experiments

In order to assess the effects of the treatment with mToxin upon the cognitive functions in mice, a battery of behavioral tests was applied. We followed our previously published protocols and measured the rate of spontaneous alternations in the single 8-min session Y-maze and performances in the Morris water maze (MWM) [41]. Trial time was limited to 60 s.

All the behavioral experiments were recorded using a Panasonic WV-CL930 camera with a Ganz IR 50/50 infrared panel. The recorded video files were analyzed using Ethovision XT 10 software (Noldus Information Technology, Wageningen, Netherlands) by an individual blinded to the treatment schedule.

### Phospho-Proteomics and Gene-Annotation Enrichment Analyses

Kinexus Antibody Microarray (KAM-900P) analyses were performed with hippocampal lysates of mice treated with mToxin and ACSF as described in our previous studies [41, 42]. Briefly, five–six animals from each group were rapidly decapitated. The brains were carefully removed. Hippocampi from the left hemispheres were used for microarray analysis. Right hippocampi served for western blotting to validate the proteomics data.

Pooled lysate proteins (100 μg) from each group were covalently labeled with a proprietary fluorescent dye combination. Free dye molecules were then removed at the completion of labeling reactions by gel filtration. After blocking non-specific binding sites on the array, an incubation chamber was mounted onto the microarray to permit the loading of two samples (one mToxin treated, and one ACSF treated) side by side on the same chip and prevent mixing of the samples. Following sample incubation, unbound proteins were washed away. Each array produced a pair of 16-bit images, which were captured and subsequently analyzed. A list of proteins with substantially changed from control (CFC) levels was generated. Priority leads have been selected with CFC ≥ 90% and normalized intensity value ≥ 1500. Possible leads were selected with CFC ≥ 60% and normalized intensity value ≥ 1000.

In order to understand biological meaning behind the proteomics data, the pathway enrichment analysis was performed using the Database for Annotation, Visualization and Integrated Discovery (DAVID) v6.8. DAVID tool was applied to uncover the significance of proteomics data and identify candidate biomarkers. Additionally, the microarray data were analyzed using a web server for functional interpretation of gene lists, g:Profiler (http://biit.cs.ut.ee/gprofiler). Finally, Cytoscape software was applied for the topological analysis and network visualization of the priority genes.

### Western Blot with A11 Antibody

To assess the levels of Aβ oligomeric species, the immunoreactivity of the A11 antibody in hippocampal lysates was examined as described previously [41]. Briefly, the hippocampal lysates from the phosphor-proteomics assay were analyzed using a Kinetworks™ Custom Multi-Antibody screen 1.0 (Kinexus Bioinformatics, Vancouver, Canada) in accordance with the instructions of the manufacturer. The analysis involves resolution of a lysate sample by sodium dodecyl sulfate-polyacrylamide gel electrophoresis and subsequent immunoblotting using validated antibodies NN198-1 (A11) and AB-CN001-1 (beta-actin). The antibodies bound to their target antigen on the nitrocellulose membrane were detected using an enhanced chemiluminescence detection system.

### Western Blotting with p-ERK1/2 Antibody

Right hippocampi were homogenized in a tissue homogenate buffer containing 50 mM Tris-HCl (pH 7.5), 150 mM KCl, 0.32 M sucrose, and protease inhibitor cocktail (10 μl for each 1 ml of lysis buffer, Sigma). Protein concentration was determined with Bradford reagent (Sigma, St. Louis, MI, USA). Six samples (20 μg) from each group were subjected to SDS-PAGE and transferred onto a nitrocellulose membrane. The membrane was blocked for 1 h in 1×PBS with Tween 20 and 5% non-fat milk followed by overnight incubation in 5% bovine serum albumin (BSA) with primary antibodies: anti p-ERK1/2 (1:1000), Hsc70 (1:3000). Following washing, the membranes were incubated with dye-conjugated secondary antibody (LI-COR Biosciences) for 1 h. Membranes were then scanned on the Odyssey CLx scanner (LI-COR Biosciences).

### TUNEL Assay

The TUNEL methodology involves the separation of fragmented, low molecular weight DNA from unfragmented, high molecular weight DNA in a given cell population. Accordingly, individual apoptotic cells are microscopically recognizable due to the characteristic appearance of nuclear chromatin condensation and fragmentation.

After behavioral tests, four mice (each group) were deeply anesthetized with pentobarbital and transcardially perfused with PBS (pH 7.4) and, subsequently, with 4% paraformaldehyde in PBS. Brains have been collected, fixed with 4% paraformaldehyde overnight, and cryoprotected in 30% sucrose in 0.1 M PBS for 3 days, which was followed by rapid freezing in −75 °C isopentane.

The brains were sliced on a Leica (Wetzlar, Germany) CM3050 S cryostat to produce 25-μm thick floating sections. The assay was carried out using an “In Situ Cell Death Detection” kit (Roche, Indianapolis, IN, USA), according to the manufacturer’s instructions. The labeling of DNA fragments was performed on coronal brain sections through hippocampi. Subsequently, the sections were counterstained with 4′,6-diamidino-2-phenylindole (DAPI).

Four sections per mouse (1.8–2.0 mm posterior to bregma) were used for the quantitative analysis. The serial sections were cut at 20-μm intervals throughout the brain. The slides were viewed under an Axio Scan.Z1 (Zeiss, Oberkochen, Germany) fluorescent scanner with a × 40/0.95 objective. The analysis was carried out on the plane-matched coronal sections. TUNEL-positive objects have been detected with Zen Blue 2.5 (Zeiss, Oberkochen, Germany) software in the dentate gyrus circle area with surface of 0.07 mm^2^ and relative positivity was calculated and presented as a bar-chart.

### β-Amyloid, 1–16 (6E10) Antibody Staining

The brain sections were blocked for 1 h in blocking solution (10% horse serum, 0.3% Triton and 1XPBS), and then incubated with primary antibodies 6E10 (1:100, Biolegend) overnight at 4 °C. The following day, the slices were washed with PBS and then incubated for 1 h with secondary antibodies Alexa 555 (1:200, Invitrogen) at room temperature for 1 h and DAPI (1:5000, Sigma) for 3 min. Following this, the slices were carefully transferred to the slides and mounted with Shandon Immu-Mount™ solution from Thermo Scientific (Cat: 990402). 6E10-positive objects have been detected in the hippocampal circle area with a surface of 0.2 mm^2^ with Zen Blue 2.5 (Zeiss, Oberkochen, Germany) software. Only dense-core Aβ plaques with a diameter of 10 ± 5 μm have been analyzed, and relative plaques density was calculated and presented as a bar-chart.

### Statistical Analysis

GraphPad Prism 8.0.1 (GraphPad Software, La Jolla, CA, USA) was used for statistical analyses. All results are presented as means with standard error. The row data were tested for normality with the Shapiro-Wilk test. To compare treatment groups of each protocol, a two-way analysis of variance (ANOVA) was used. Bonferroni’s test was used for multiple comparisons. One-way ANOVA with Tukey’s multiple comparisons or repeated-measures ANOVA were used to analyze ex vivo data. The significance of the difference between the experimental groups in western blotting and TUNEL assays was determined using Student’s *t* test. *P* value <0.05 was considered statistically significant.

## Results

### Characterization of Modified Snake α-Neurotoxin

**Fig. 1:**
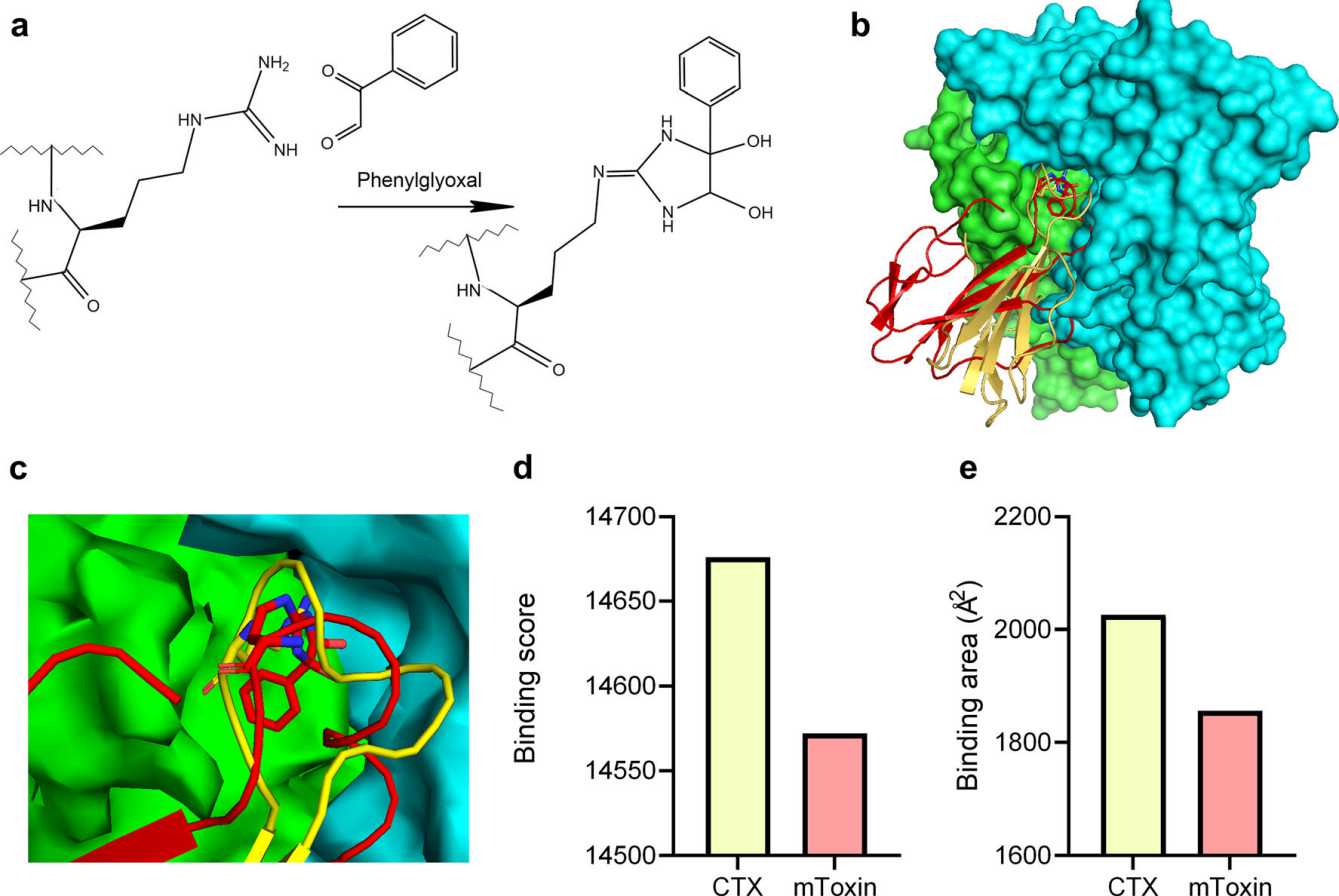
**a** Reaction of phenylglyoxal with the arginine moiety in peptides yields imidazolidine diol. **b** Overview of CTX (yellow) and mToxin (red) binding to the complementary acetylcholine-binding protein (AChBP). Chains A and B of AChBP are colored in green and blue respectively (PDB ID: 1YI5). **c** A closeup of A and B subunits’ ACh binding site with modified CTX and natural CTX. **d** The binding scores of the top docking results for CTX and mToxin, accepted by PatchDOCK. **e** The binding areas of the CTX and mToxin with the receptor, accepted by PatchDOCK

Purified α-cobratoxin (CTX) was modified with phenylglyoxal following a previously published protocol [29]. The reaction (Fig. 1a) resulted in a stable final product, which was tested via MALDI. The mass spectrum of the product displayed a distinctive peak at 8397 Da (Supplementary Fig. 1) corresponding to the expected mass of the fully modified α-cobratoxin in which five arginine molecules are substituted with phenylglyoxal following the release of five molecules of waters and protons. (CTX—7821 Da, five molecules of phenylglyoxal (C_8_H_6_O_2_, 134.2 Da)—671 Da, five molecules of water (H_2_O, 18 Da)—90 Da, five protons (H^+^, 1 Da)—5 Da). Therefore, the chemical reaction with phenylglyoxal successfully modified CTX in accordance with our prediction. All five arginine residues are substituted with phenylglyoxal molecules.

### In Silico Binding Modeling of CTX and mToxin to AChR Reveals Similar Interaction Modes

PatchDOCK software predicts protein-protein interactions and calculates the buried surface area and the binding score upon complex formation.

PatchDOCK algorithm predicts a similar binding position of unmodified and modified CTX (mToxin) in the α7ACh-like receptor binding site (Fig. 1b, c). However, modification of CTX considerably reduces the predicted binding score from 14,676 (CTX) to 14,572 (mToxin) (Fig. 1d). Similarly, modification of CTX also reduces (from 2026 to 1856 Å^2^) the buried surface area formed upon complex formation with α7ACh-like receptor (Fig. 1e). Both the binding score and the buried surface area are widely accepted in the literature to estimate the binding affinity [43].

Interestingly, the highest score of PatchDOCK corresponded to CTX in the correct orientation, while only the second highest score of PatchDOCK corresponded to mToxin the correct position.

The results indicate that mToxin could serve as minimally toxic inhibitor that negligibly affects receptor function; however, it still prevents the Aβ binding via competitive mechanisms.

### mToxin Moderately and Reversibly Inhibits α7AChR at Nanomolar Range

**Fig. 2:**
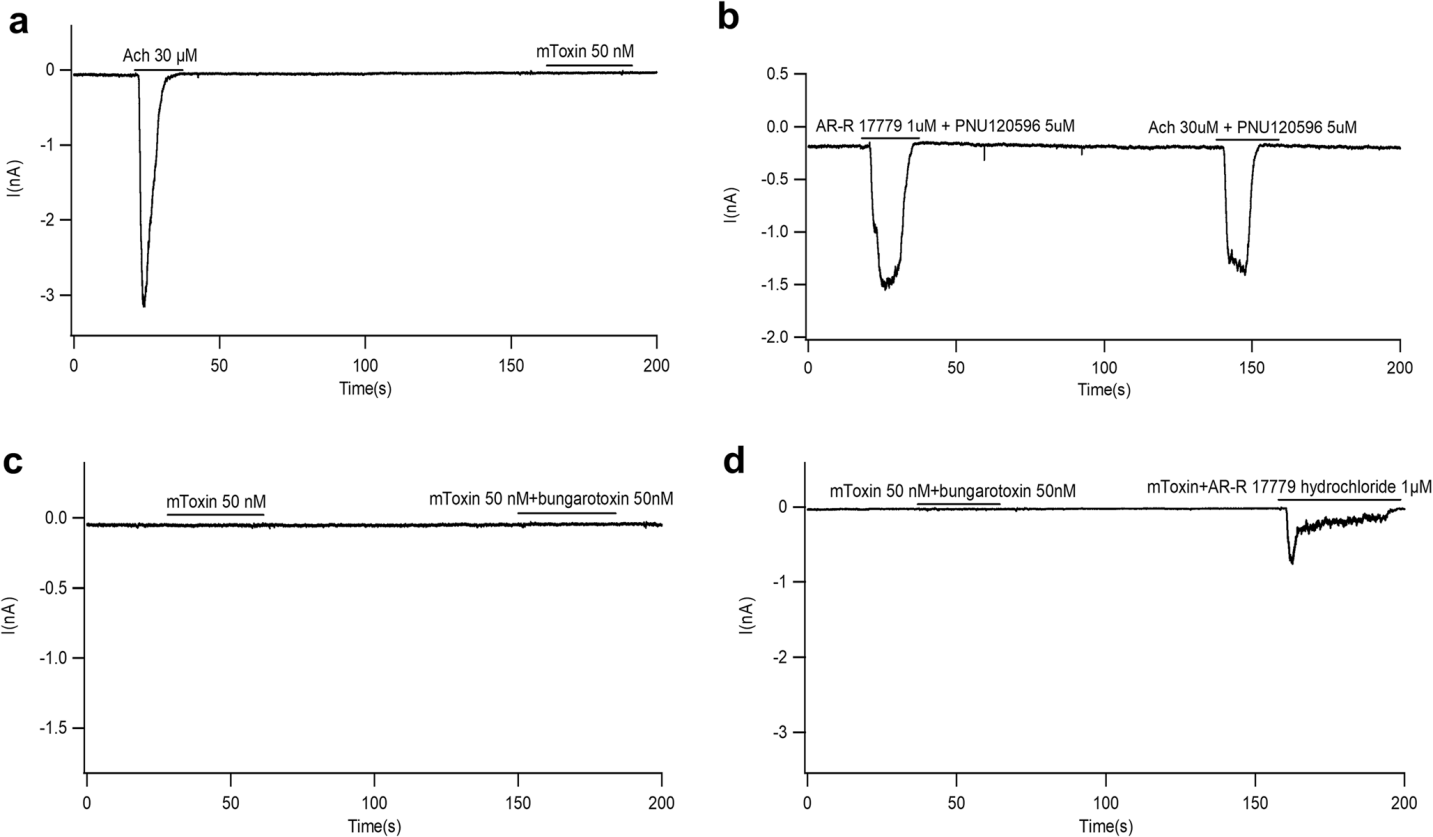
Representative whole-cell recordings from PC-12 cells stimulated with various agents. **a** ACh (30 μM) induces a typical brief response. Application of mToxin does not induce any response. **b** Potent selective α7AchR agonist AR-R 17779 (1 μM) mimics ACh (30 μM) and induces a similar response in the presence of the positive allosteric modulator of α7AchR, PNU120596. Note the overwhelming response of α7AChR, but not of α1AChR. **c** Natural toxin (α-bungarotoxin) and mToxin do not induce any current in PC-12 cells. **d** mToxin reduces (by about 75%) the amplitude of AR-R 17779-induced currents. Note, that mToxin is a partial antagonist of α7AChR

We took advantage of the whole-cell patch-clamp recording methodology to investigate mToxin effects upon α7AChR. The ACh application (30 μM) served as a reference signal for a more efficient comparison between experiments (Fig. 2a). All responses were recorded in the presence of positive modulator PNU-120596 (5 μM) (Fig. 2 b).

Homomeric α7AChR are cation permeable and are efficiently blocked by bungarotoxin. Previous studies established that the application of 50 nM of natural α-bungarotoxin is sufficient to completely abolish synaptically evoked action potentials mediated by α7AChR [44]. Moreover, nanomolar concentrations of CTX and α-bungarotoxin block in irreversible manner the nicotinic synaptic response in the hippocampus [45, 46].

We modified CTX and tested its ability to block α7AChR-mediated currents. First, we examined the reaction to mToxin (50 nM) without agonist. mToxin alone and in combination with unmodified α-bungarotoxin did not induce any current in PC12 cells (0.00%±0.00%) (Fig. 2a, c). Application of the specific α7AChR agonist AR-R 17779 (1 μM) induced a current response similar to the observed with 30 μM of ACh (121.6 ± 24.97%) in the presence of the positive allosteric modulator of α7AChR, PNU120596. At these concentrations, AR-R 17779 mimic ACh, and concur with the assumption that the vast majority of acetylcholine receptors in the hippocampus are α7AChR, and not α1AChR. (Fig. 2b). Remarkably, mToxin (50 nM) did not fully block the AR-R 17779 (1 μM) induced currents; though, significantly reduced its amplitude (25.93±9.66%, *p*=0.002) relatively to ACh (Fig. 2d). Most noteworthy, (in contrast to natural α-neurotoxins), mToxin partially inhibits α7AChR-associated currents, just for a brief period of several seconds.

To quantify the α7AChR-mediated currents in relation to mToxin application as a function of time, we calculated the activation-inactivation rates of the receptor (Table 1).

**Table 1:** Activation-inactivation modes of PC-12 cells reaction to ACh, AR-R 17779, and mToxin application

ACh		AR-R		mToxin+ AR-R	
Aτ_1/2_ (s)	Dτ_1/2_ (s)	Aτ_1/2_ (s)	Dτ_1/2_ (s)	Aτ_1/2_ (s)	Dτ_1/2_ (s)
1.1±0.25	1.75±0.68	0.72±0.11	1.77±0.13	0.69±0.16	6.56±0.33

Of note, half-activation (Aτ_1/2_) is the time during which the current via the channel reaches half of its peak value. Half-inactivation (decay period) reflects the receptor desensitization rate and represents the time during which the current drops to half of its peak values (Dτ_1/2_) [47]. The latter reflects the acceleration rate of the transition to the receptor’s closed state.

Table 1 suggest that mToxin (50 nM) application does not lead to significant changes in the channel activation pattern by AR-R 17779; however, it substantially (by about fourfold) extends the decay period (*n*=4 for each group, mean values (seconds)±SEM). mToxin may stabilize the open conformation of α7AChR, hinder channel closure, and prevent receptor desensitization.

Taking together, these results point to mToxin weak and reversible inhibitory properties at the nanomolar range. Its application moderates the ACh-dependent current via the channel, reduces its amplitude, and prolongs the duration.

### mToxin Rescues Aβ-Induced Impairment of Long- and Short-Term Synaptic Plasticity in Ex Vivo Model of Alzheimer’s Disease

In light of our in vitro results, and in order to investigate the influence of mToxin on presynaptic and postsynaptic mechanisms of neuronal plasticity, we utilized two canonical ex vivo paradigms. We used brain slices of wild-type animals to measure paired pulse facilitation (PPF) ratios and long-term potentiation (LTP) of extracellularly recorded field excitatory postsynaptic potentials (fEPSP), which reflect accordingly the processes of short- and long-term neuronal plasticity. Electrophysiological studies were conducted under various conditions, and measurement was carried out after pre-incubation with ACSF (control), mToxin, Aβ, and mToxin with Aβ.

**Fig. 3:**
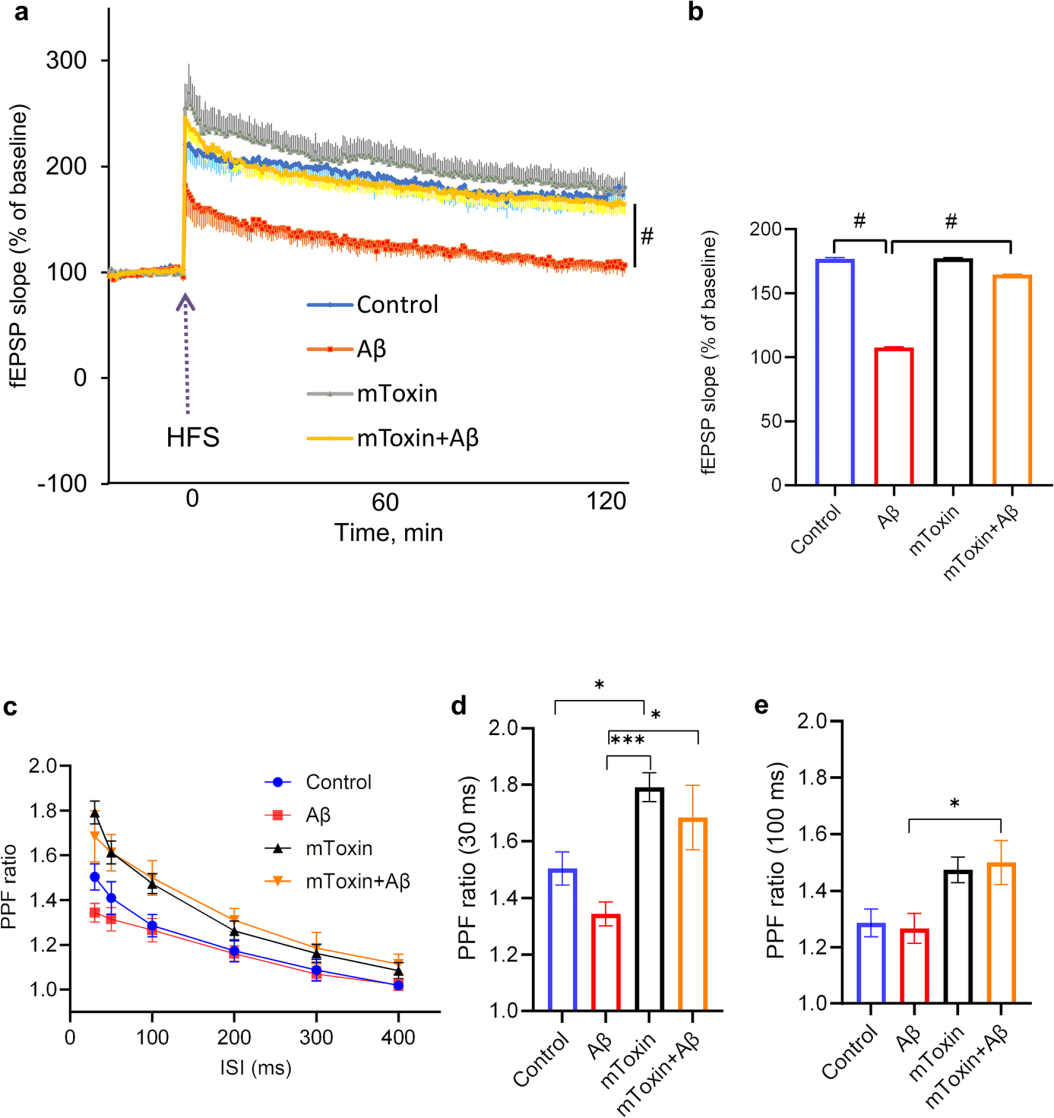
Improvement of basal synaptic transmission and rescue of hippocampal LTP with mToxin treatment. **a** Hippocampal LTP in the CA1 area. Soluble aged Aβ inhibited LTP (red tracing) induced by high-frequency stimulation (HFS, arrow), and was restored by mToxin (yellow tracing, *n* = 9) to its control level (blue tracing, n = 9). mToxin alone had no significant effect on LTP (gray tracing, *n*=9). **b** Effects of Aβ and mToxin on the maintenance phase of LTP, 115–120 min after HFS. **c** PPF at different ISI with various treatments, **d** PPF ratios recorded at 30 ms ISI. **e** PPF ratios recorded at 100 ms ISI. *p<0.05, **p<0.01, ***p<0.001, #*p*<0.0001

PPFs were generated by applying two stimulation pulses separated by various interstimulus intervals (ISI) ranging from 30 ms to 400 ms. As expected, we observed an inverse correlation between the PPF ratio values and the duration of the intervals in all experimental groups (Fig. 3c). Moreover, the differences in PPF values between the groups are inversely correlated with inter-stimuli intervals (ISI). Remarkably, the slices treated with mToxin demonstrated the PPF ratio values, which are substantially greater than in the control and Aβ-treated groups. One-way ANOVA analysis was applied to compare the means and revealed that the differences are statistically significant in the groups with ISI of 30, 50, and 100 ms. The most significant difference has been observed in the group of 30 ms interval. The main effect of the type of treatment had *F*_(3, 32)_=7.445 with *p* value=0.0006. Tukey’s multiple comparisons test proved that mToxin treatment significantly (adjusted *p* value=0.0399) improved the PPF ratio by ~19% compared to ACSF (control) treatment from 1.50±0.05 to 1.79± 0.05 (Fig. 3d). Aβ-treatment expectedly depressed the PPF ratio values; however, the application of mToxin rescued the deteriorating effect of Aβ from 1.34±0.04 to 1.68±0.11 (adjusted *p* value= 0.0115). With long ISI, the main effect of the treatment upon PPF values declined; however, it was still significant in the experiment with ISI of 100 ms (*F*_(3,32)_=4.502; *p* value=0.0096). Multiple comparisons test revealed a significant difference (*p*˂0.05) only between the means of Aβ-treated and Aβ+mToxin treated groups (Fig. 3e). These results indicate that mToxin improves synaptic plasticity via presynaptic mechanisms and diminishes the Aβ-associated deteriorating effect upon it.

To examine the long-term plasticity, LTP was recorded after four 100-Hz trains spaced 5 min apart in hippocampal slices under the same conditions. Pre-incubation with Aβ peptide (50 nM) resulted in a typical substantial impairment of LTP. These data accord with the current literature [48] and with our previously published results obtained in the same paradigm [36]. Likewise, there was no change in baseline responses within all the tested groups, which is in line with previous studies proving that Aβ has no effect on normal synaptic functioning on a baseline level [49]. Remarkably, pre-incubation of slices with a mixture of Aβ peptide and mToxin did not lead to significant changes in E-LTP (252±13%, *p*=0.79) compared to control recording from slices treated with ACSF (Fig. 3a).

The recording continued for 2 h post HFS to assess the maintenance phase of LTP. Repeated measures ANOVA revealed a very significant (*p*˂0.0001) difference between the group treated with Aβ and mToxin+Aβ (Fig. 3a). The detailed comparison between all treated groups with one-way ANOVA proved a significant effect of the treatment upon the late-phase LTP (Fig. 3b). Of note, LTP recordings from female brain slices demonstrated very similar patterns (Supplementary Fig. S2).

These results point to the multifaceted effects of mToxin on neuronal activity. HFS triggered a characteristic E-LTP beginning right after the stimulation and depending upon calcium dynamics and kinases activity. We applied a relatively strong stimulation protocol with four consequent 100-Hz trains to induce LTP that lasts for several hours and depends on de novo gene transcription [50]. Evidently, mToxin effects are sufficient to cope with Aβ-induced toxicity and rescue the deteriorating effect of Aβ peptide on long-term synaptic plasticity, which suggests a protein synthesis-dependent mechanism.

One recent elegant study in a rat model by Goethem et al. (2019) demonstrated that α7AChR antagonists are capable of potentiating receptor function, increasing glutamate efflux, and enhancing hippocampal LTP [51]. Our data accord with these findings.

### Modified Toxin Demonstrates No Toxicity in Mice

**Fig. 4:**
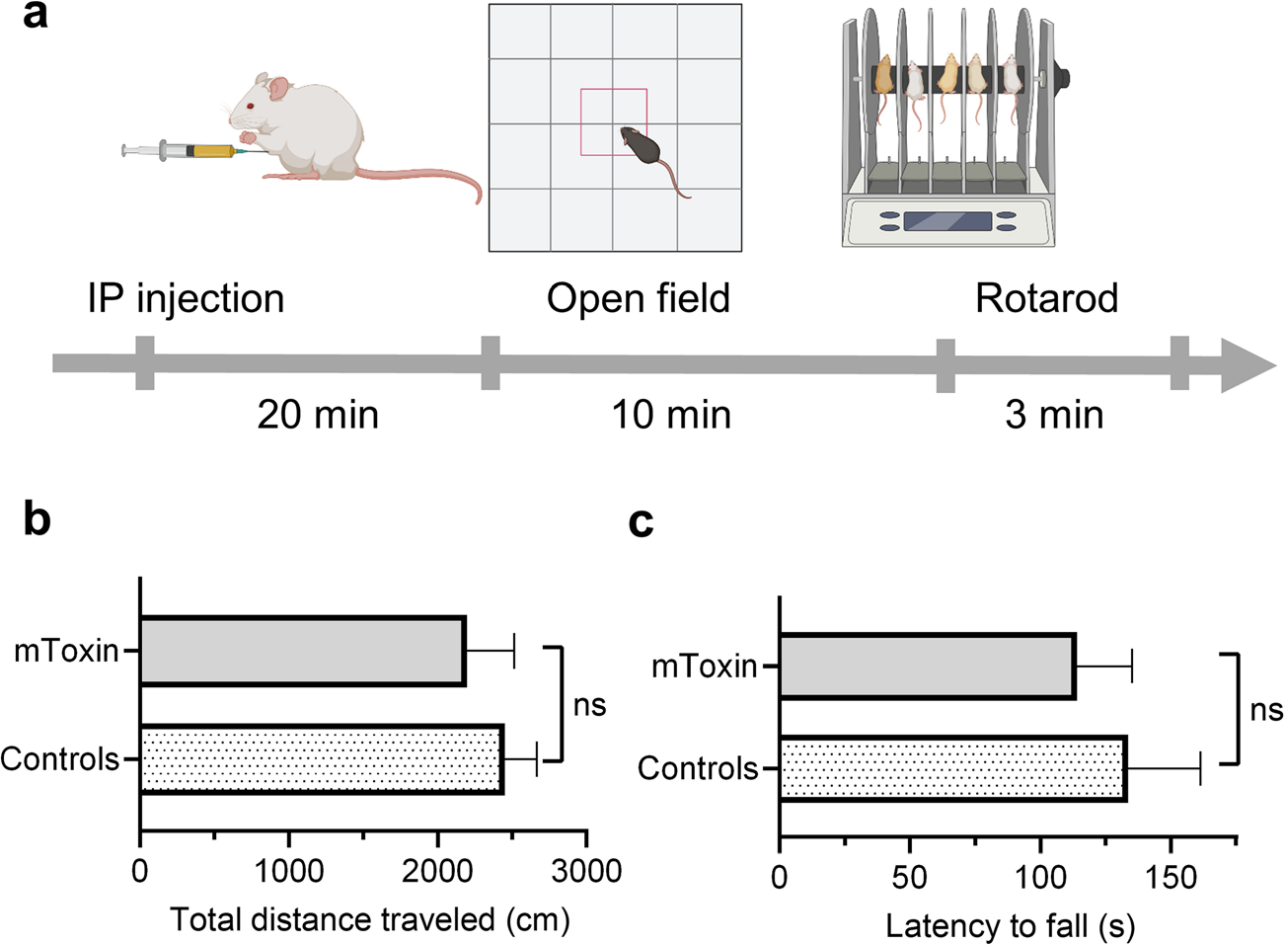
mToxin toxicity test in mice. **a** Experimental design and timeline of the procedures. Animals were injected IP and tested in open field maze and rotarod. **b** The mice traveled the same distances in open field maze. **c** Latencies until fall from the rotarod are very similar in both experimental groups. (*n*=4)

CTX is an extremely toxic venom with LD50 of about 0.1 mg/kg [38]. Several decades ago, Yang et al. proved that the chemical modification of CTX with phenylglyoxal substantially reduces its lethal and antigenic activity [47]. In this study, we assessed the effects of mToxin on locomotion in mice (Fig. 4). We administered the substance intraperitoneally in a dose, which should lead to 100% lethality if it were a natural venom (Fig. 4a). Remarkably, all animals survived the experiment. Moreover, the procedure did not lead to observable effects in the animals’ appearance or behavior. The mice injected with mToxin demonstrated the same behavioral patterns as the animals injected with PBS. We measured the total distances traveled by the mice in open field maze. The Mann-Whitney test did not reveal a significant difference between the treatment groups (*p*=0.3429) (Fig. 4b). Subsequently, the animals were subjected to a rotarod test, which also revealed no differences (Fig. 4c).

### Intracerebroventricular Administration of mToxin Has No Effect on Memory Acquisition and Recall in Wild-Type Mice, but Improves Short-Term Memory in Alzheimer’s Disease Mice

In order to determine the effects of mToxin on learning and memory in wild-type animals and in AD murine model, we administered the mToxin intracerebroventricularly and analyzed the mice performances in various paradigms.

**Fig. 5:**
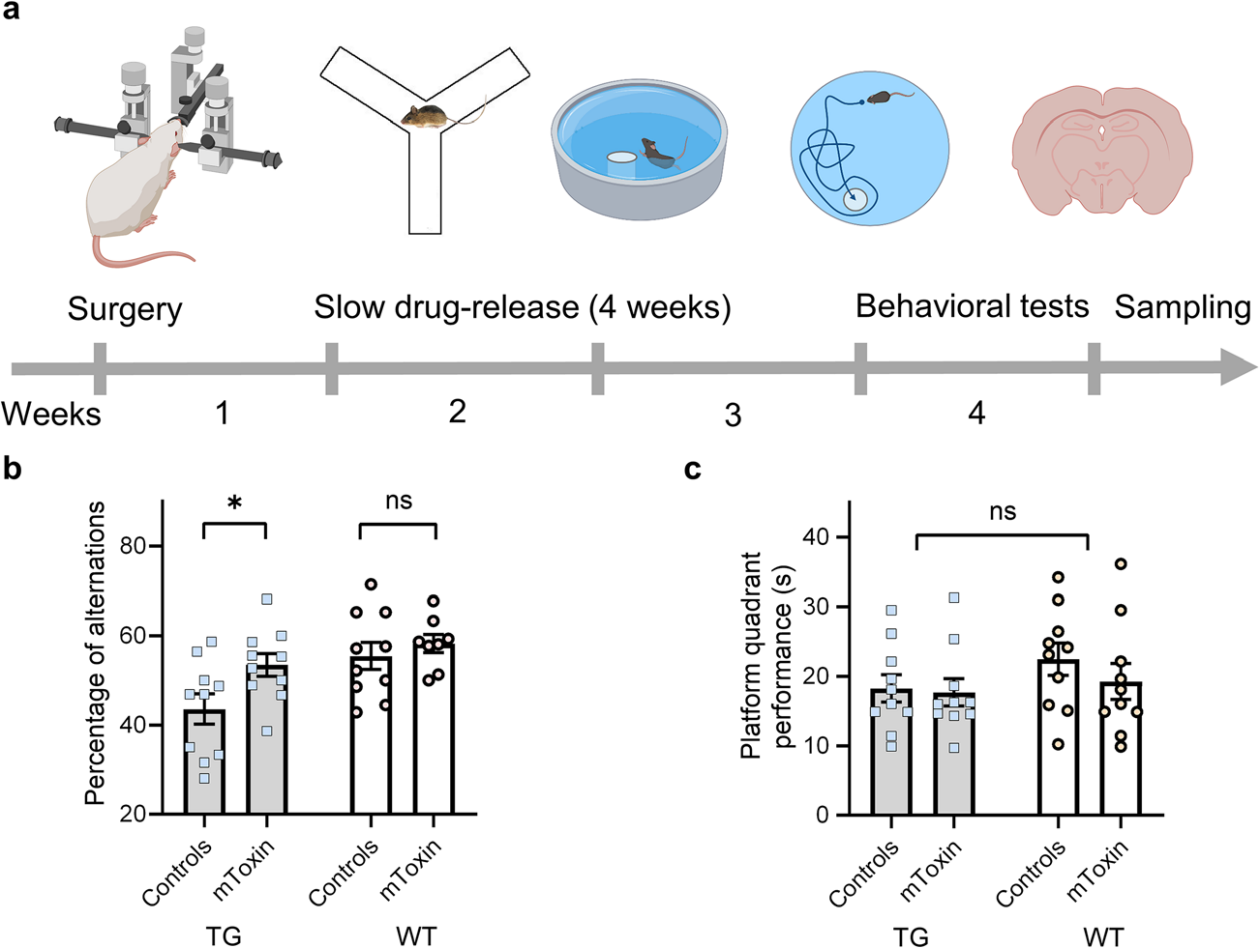
Schematic representation of experimental design with a depiction of a chronological timeline. **a** Behavioral tests and tissue sampling followed stereotactic surgery with intracerebroventricular canulae implementation. **b** The Y-maze test results are presented as the percentage of spontaneous alterations made by the animals in a single 8-min-long session. **c** The time spent by the mice in the target quadrant during the probe trial of MWM. *p value<0.05, *n *= 10. TG-3×Tg mice, WT-wild type mice

We used Y-maze to assess spatial working memory in relation to the treatment and genotype. We observed significant improvement in the rate of alternations in transgenic mice treated with mToxin (*p*=0.036). Wild-type animals performed significantly better than transgenic animals (*p*=0.0068); however, the treatment had no effect within this group (Fig. 5b). Moreover, interaction accounts for just 3.1% of the total variance with *p*=0.23, meaning that there is no interaction effect between treatment and genotype.

We continued behavioral studies with MWM, which is one of the most widely used tests in behavioral neuroscience. The mice were trained to find a hidden platform for five consequent days. Wild-type animals performed expectedly better than the transgenic mice, which accords with our previous results [41]. Nevertheless, repeated-measures ANOVA did not reveal a significant effect of the treatment upon the rate of memory acquisition within the groups (data not shown). Furthermore, during the test phase, there was no significant effect (*p*=0.4) of the treatment upon time spent in the platform quadrant (Fig. 5c).

### mToxin Attenuates Aβ-Driven Apoptosis in a Rodent Model of AD

A substantial acceleration of apoptosis and progressive neuronal cell death are distinctive characteristics of AD pathogenesis [52], which have been consistently replicated in murine models, including 3×Tg mice [53]. Notably, the apoptotic index has been shown to be responsive to various treatments. We used a standard apoptotic marker TUNEL assay to assess the mToxin effects on the rate of apoptosis in the AD mice hippocampi. TUNEL detects DNA degradation via enzymatic incorporation of labeled dUTP into free 3′-hydroxyl termini created as a result of DNA fragmentation. Therefore, it is a relatively sensitive and simple method [45].

**Fig. 6:**
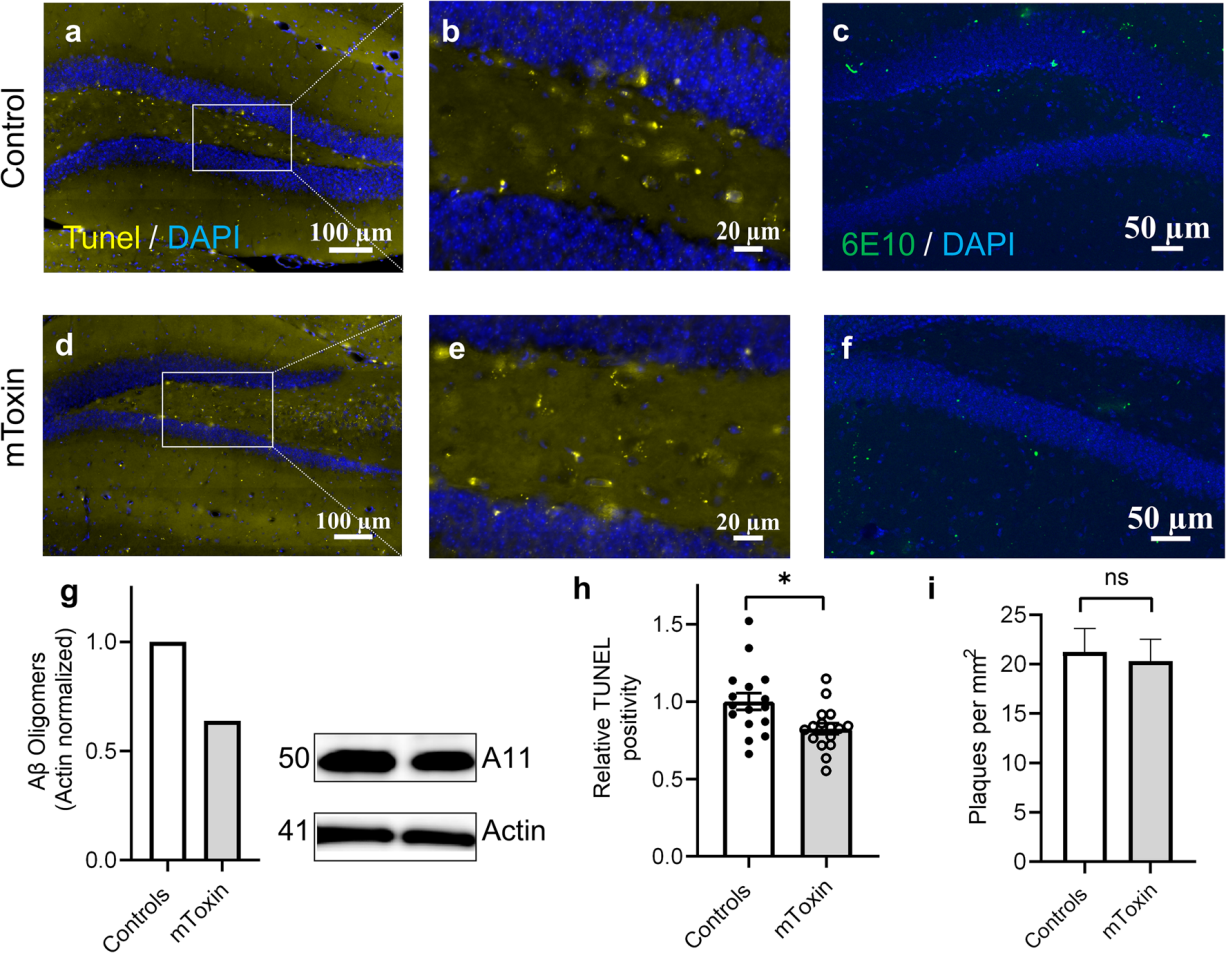
**a, d** TUNEL staining in the hippocampi of 3×Tg mice (40×magnification). **b, e** Dentate gyri digitally zoomed-in. **c, f** 6E10 staining of 3×Tg mice coronal sections detects Aβ plaques of various dimensions (green objects). **g** Kinetworks™ Custom Multi-Antibody screen 1.0 western blot of pooled lysates (four animals each group). **h** Relative TUNEL-positive surface area (unpaired two-tailed t-test, *p<0.05). **i** Amyloid-β plaques’ density in the hippocampi of 3×Tg mice (t-test) *n*=16, four mice

We analyzed the TUNEL positivity in the hippocampal dentate gyri (Fig. 6 a, d). The quantities of fragmented DNA were diminished following the treatment with mToxin. Detailed analysis of the apoptotic cells in the dentate gyrus (Fig. 6b, e) revealed a treatment-associated significant effect (*p*=0.013) upon the relative surface area of TUNEL-positive objects (Fig. 6h).

### mToxin Diminishes the Quantities of Aβ Prefibrillar Oligomers but Not Plaques in the Hippocampi of the 3×Tg Mice

Most of the neurodegenerative diseases are characterized by the brain accumulation of protein aggregates. Recent evidence indicates a crucial role of soluble amyloid oligomeric species in synaptic dysfunction, neuronal apoptosis, and brain damage associated with AD development [46]. To further explore the impact of mToxin upon AD pathogenesis, we tested the amount of toxic Aβ oligomeric forms in the hippocampal lysates via traditional immunoblotting methodology. Remarkably, the treatment resulted in a substantial 36% reduction in the levels of A11-reactive oligomers (Fig. 6g).

We also pursued the detailed amyloid burden examination within the brains of 3×Tg mice in relation to the treatment and stained the coronal sections with human APP/Aβ-specific antibody. The staining procedure was calibrated to detect extracellular diffuse and dense amyloid plaques. Of note, eight-month-old 3×Tg mice begin to display characteristic Aβ extracellular deposition [39]. Several plaques have been observed in the hippocampi. We analyzed only dense-core Aβ plaques with a diameter of 10 ± 5 μm, which are the most common in AD mice [54]. The assay did not detect significant differences in the amyloid plaques’ density index between the experimental groups (Fig. 6i).

### mToxin Induces Neuronal Plasticity-Related Biological Pathways in AD Mice

Under normal conditions, α7AChR activation by low concentrations of Aβ triggers signal transduction cascades associated with synaptic plasticity, neuroprotection, learning, and memory. It was suggested that the MAPK pathway is mainly responsible for this phenotype [55].

In order to examine the effects of mToxin at the molecular level in vivo, we utilized an advanced antibody microarray assay and western blotting of hippocampal lysates. The assay revealed dozens of proteins with substantially increased or decreased levels following the treatment with mToxin. Of these, 92 were determined as priority leads with ≥90% change from control (ACSF treated) and 96 as possible leads with ≥60% change (Supplementary Table S2).

β-Catenin is the protein with the most substantially amplified (by 434%) treatment-associated levels. Of note, β-catenin is a dual function protein involved in regulating cell adhesion and gene transcription. Moreover, it is strongly implicated in neuronal synapse regulation and remodeling and is required for memory consolidation [56]. In addition, our assay detected an increase of 349% in the protein levels of dual specificity mitogen-activated protein kinase 1 (MAP2K1), which integrates multiple biochemical signals. This kinase is situated upstream of MAP kinases and stimulates their activity upon activation by various stimuli. As an essential component of the MAP kinase signal transduction pathway, it is involved in cellular proliferation, differentiation, and development. Accumulating evidence points to a central role for translational control by MAPK signaling in long-lasting forms of synaptic plasticity and memory acquisition [57]. Another central kinase, dual-specificity MAPK/ERK protein-serine kinase 2 (MAP2K2), increased by 174% in levels following the treatment. MAP2K2 is required for autophagy-associated clearance of pathological proteins, including Aβ, in neurodegenerative diseases [58]. The upregulation of MAP2K2 kinase activity has been shown to induce neuroprotective autophagy.

**Fig. 7:**
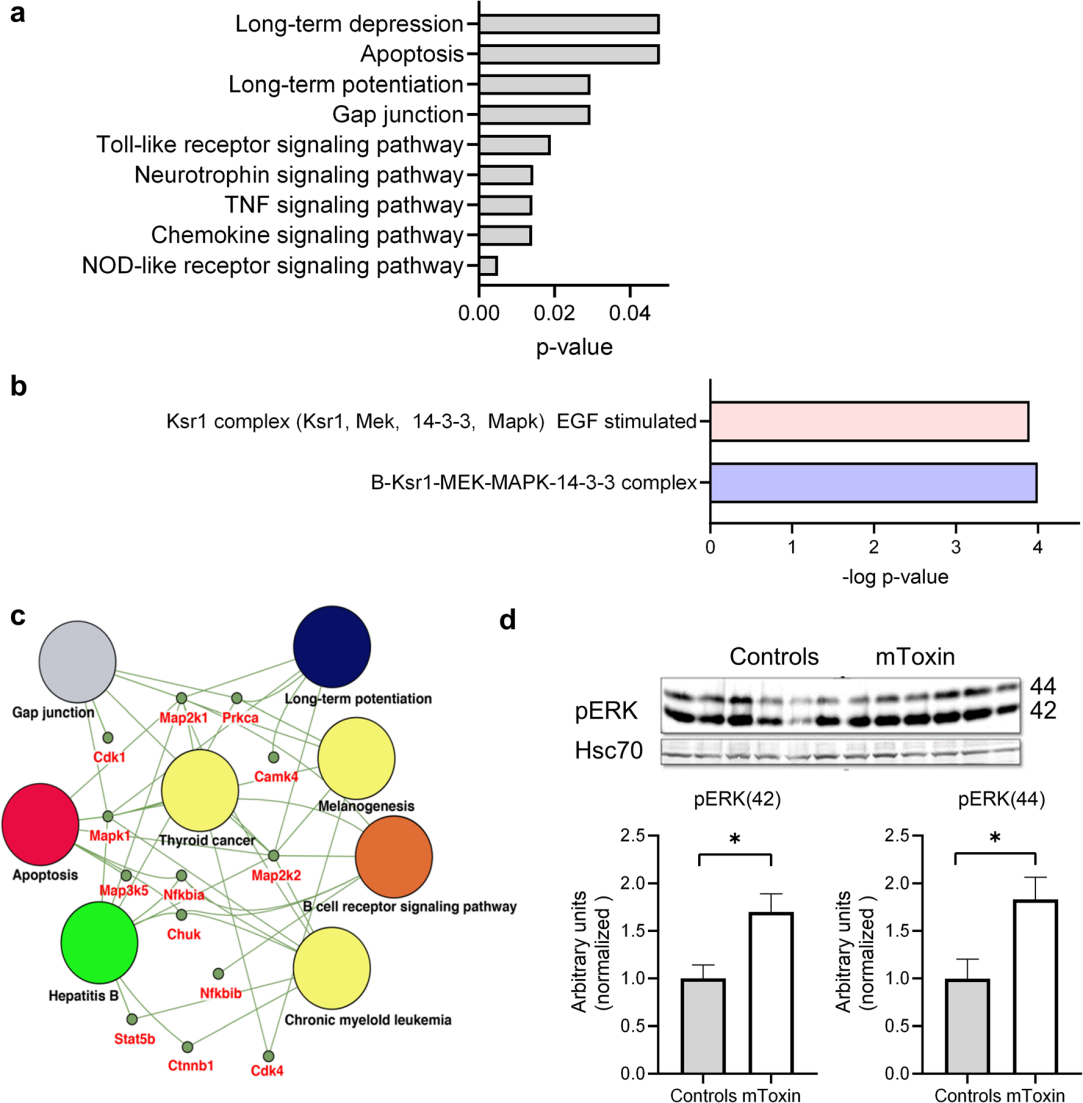
**a** g:Profiler pathway enrichment analyses with the KEGG database. **b** CORUM protein complexes known for annotated protein complexes revealed enrichment for B-Ksr1-MEK-MAPK-14-3-3 complex and Ksr1 complex (Ksr1, Mek, 14-3-3, Mapk), EGF stimulated. These protein complexes are enriched for MAPK cascade and neurogenesis. **c** Protein-protein interaction network and functional enrichment for the KEGG pathway with priority leads were visualized in Cytoscape. It presented the enrichment results with nodes representing gene-sets and edges representing protein-protein associations. Accordingly, these proteins contribute to a mutual function; nevertheless, they are not necessarily physically binding to each other. **d** Western blot analysis of hippocampal lysates of and mToxin treated mice using p44/42 MAPK (Erk1/2) antibody and Hsc70 antibody. *N*=6, *p<0.05 *t* test

Of note, MAP2K2 phosphorylates and consequently activates extracellular regulated kinases (ERK1 and ERK2). Our phospho-proteomics assay revealed that ERK1 phosphorylated at Y204 demonstrated a 178% increase following the treatment. We validated the phospho-proteomics data acquired by analysis of pooled tissue samples and tested the levels of pERK via a standard western blot assay, which confirmed the direction of change (Fig. 7d). Immunoblotting with antibodies against p44/42 Erk1/2 showed about 80% significant increment in the brains treated with mToxin.

We used the g:Profiler web server and DAVID tool to determine the functional annotation of the leads and uncover the significance of phospho-proteomics data. Using the Kyoto Encyclopedia of Genes and Genomes (KEGG) library, we found that leads were particularly enriched in pathways related to synaptic plasticity, cell signaling, and inflammation (Fig. 7a). Bioinformatics analysis of protein-protein interaction with the CORUM protein complexes annotation database proved a significant MAPK cascade implication in the molecular machinery responsible for the observed phenotype (Fig. 7b). The Cytoscape App served to visualize the gene-set enrichment results as a network enrichment map (Fig. 7c).

Of note, together with an increment, the treatment with mToxin led to a substantial decline in levels of a list of proteins. Several of them are strongly related to neuroinflammation and astrogliosis. For instance, glial fibrillary acidic protein (GFAP) phosphorylated at S8 demonstrated a 78% decline (supplementary Table S2). GFAP is a protein highly expressed in astrocytes that is involved in cell communication and the functioning of the BBB. Human postmortem studies report increased quantities of phosphorylated GFAP in AD brains [59]. This suggests that mToxin reduces the rate of Aβ-driven astrogliosis, which is a characteristic feature of AD [60].

## Discussion

For several decades, Aβ has been considered as the key AD pathogenetic factor triggering complex downstream events, which eventuate in synaptic dysfunction, neurodegeneration, and clinical dementia [1]. Obviously, this simplistic view is not adequate to explain all the AD-associated phenomena and must be reconsidered.

One of the most intriguing scientific questions to be addressed is the dual nature of Aβ activity in the brain. Indisputably, APP is essential for memory formation in mammals [61]. Moreover, accruing empirical evidence indicates that in a healthy brain, an APP derivative, Aβ protein, mediates learning and memory. It has been shown in a murine model that hippocampal injection of picomolar concentrations of exogenous Aβ improves memory consolidation [62]. Accordingly, it was suggested that Aβ acts differently in the brain under different conditions. So, physiological picomolar Aβ content supports essential functions in the healthy brain, although in AD patients with escalated Aβ levels, it interferes with normal synaptic function, prompts the formation of plaques, and eventuates in cognitive decline.

**Fig. 8:**
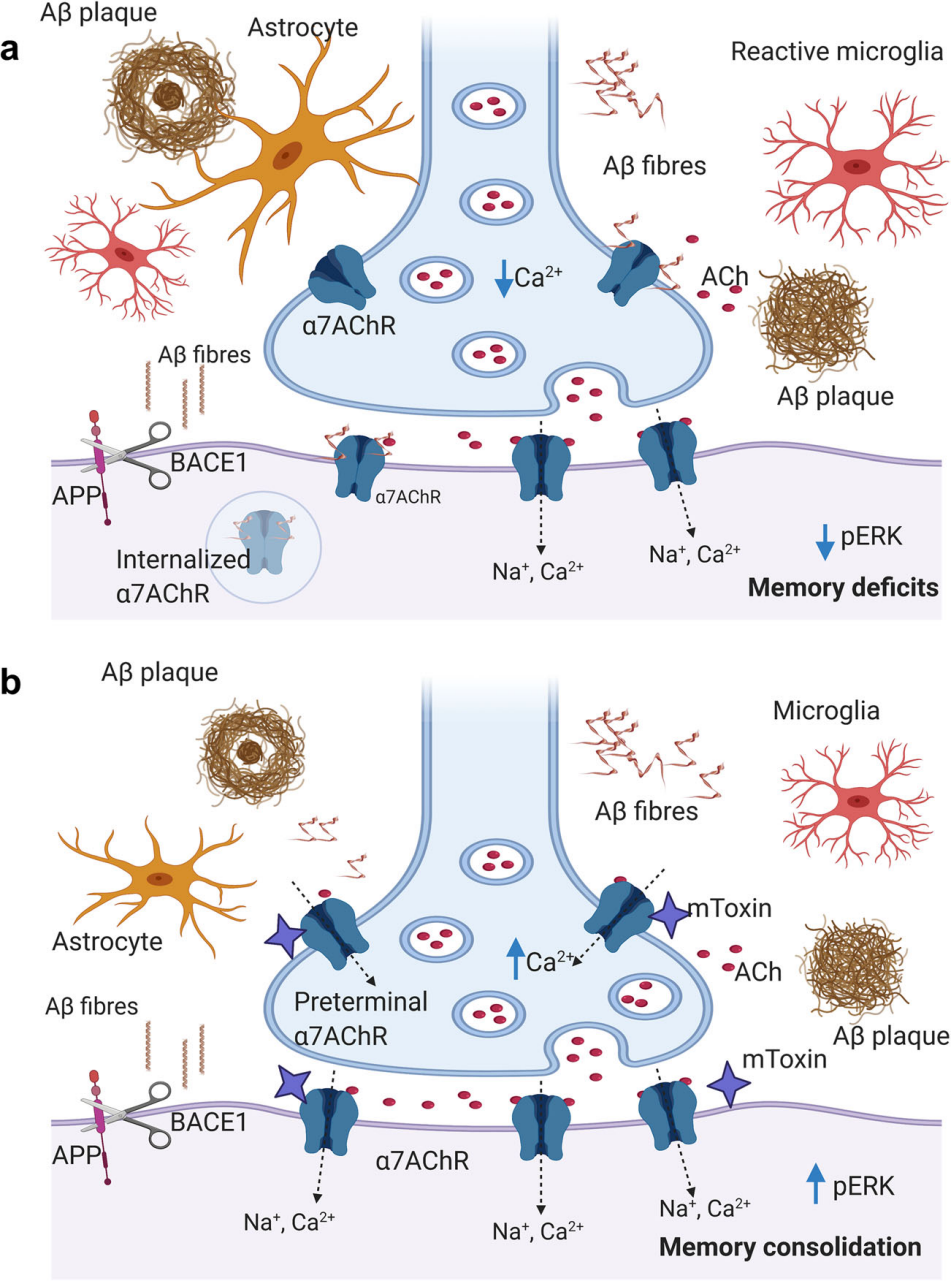
Proposed model of mToxin effects in the brain of AD mice (with emphasis upon α7AChR function). **a** The development of AD-like phenotype in mice is characterized by neuroinflammation, astrogliosis, gradual Aβ deposition, cholinergic deficits due to block of α7AChR by Aβ oligomeric and fibrillar species, followed by α7AChR internalization. Also, these processes eventuate in the downregulation of the MAPK/ERK pathway that exacerbates the cognitive decline. **b** mToxin competes with Aβ for the binding to α7AChR and modulates its activity. Also, the treatment leads to a reduction in astrogliosis and neuroinflammation, which subsequently moderates the APP transcription, translation, and processing rates. Likewise, the treatment eventuates in activation of ERK and memory improvement. Of note, neuronal nicotinic acetylcholine receptors are located preterminally, presynaptically, and postsynaptically. ACh released from presynaptic vesicles diffuse and activate presynaptic α7AChR as well as postsynaptic, which results in the recruiting of neurotransmitter reserves and modulation of its release. Accordingly, PPF demonstrate significantly improved ratios following the treatment with mToxin. BACE1-β-secretase

Aβ exerts some of its functions through α7AChR to which it binds with high affinity (Fig. 8a) [21]. The mechanism and biological relevance of this interaction remain enigmatic [63]. An interesting model suggests that synaptic activity triggers the release of Aβ, which, consecutively, modulates α7AChR activity and leads to improvement in synaptic plasticity and memory acquisition [64]. Moreover, Aβ acts dualistically as an agonist or antagonist of α7AChR, depending on its concentration.

In the AD brain, elevated Aβ levels interfere with the normal function of α7AChR. Aβ–α7AChR interaction under these conditions aggravates the Aβ toxicity via weakening the α7AChR-dependent neuroprotective signaling [17], leads to Aβ-receptor complex internalization and plaque formation (Fig. 8a) [23]. It is noteworthy that α7AChR internalization following Aβ binding is preventable via application of α-neurotoxins, such as bungarotoxin (Fig. 8b) [65]. Likewise, Aβ-induced toxicity and τ-protein hyperphosphorylation are efficiently avertible by α-bungarotoxin [66]. Accordingly, natural snake venoms represent an attractive scientific model and a promising direction for drug development.

In general, α7AChR-selective antagonists are capable of blocking receptor activation by Aβ, which may prevent its seeding, nucleate deposition, and plaque formation [26]. Co-administration of nicotinic antagonists attenuates a highly potent agonist-like effect of Aβ N-terminal fragment upon nicotinic receptors [67]. Consequently, we hypothesized that targeting the Aβ-α7AChR interaction may be an efficient AD therapy. Considering recent reports, we designed an original modification of α-neurotoxin to interfere with Aβ binding to α7AChR in a competitive manner. mToxin is a derivative of α-neurotoxin, CTX, which binds to the α7AChR and inhibits Aβ binding through mimicry mechanisms [28]. As a member of the α-neurotoxins family, CTX is highly toxic in mice with LD_50_ of 0.1 mg/kg [38, 68]. In order to reduce toxicity, we rationally modified its structure and substituted all CTX arginine residues with phenylglyoxal. This methodology has been described in the literature as an approach that dramatically reduces the lethality of CTX [31].

We hypothesized that mToxin does not block AChR function; rather, it acts as a weak competitive modulator that prevents Aβ binding. First, we tested our hypothesis in silico and evaluated the binding mode of the modified toxin to α7AChR by using PatchDOCK software. The results prove that adding a bulk side group to arginine 33 partially prevents the arginine to insert into the binding pocket but allows ACh binding to the receptor and its activation.

Notably, the size of the arginine substitution could play an important role in toxicity, and we speculate that large glyoxal substitutions would further reduce the toxicity of snake toxins. Thus, by increasing the size of the arginine substitution, toxicity and binding would be reduced, until first all toxicity is lost, and later all binding is lost through finger I and the C-terminus of snake α-neurotoxins. Somewhere along this curve, an optimal modification could be identified, that protects against snake toxicity, yet does not abolish binding. Also, the choice of snake α-neurotoxins could play an important role in toxic binding, and long α-neurotoxins could bind more strongly than short α-neurotoxins lacking a C-terminal tail.

We continued our studies in a series of in vitro experiments. We revealed that mToxin interacts reversibly with α7AChR and acts as a partial antagonist, which restrains the α7AChR-related currents for a brief period of several seconds. It is well-established in the literature that nanomolar concentrations of α-bungarotoxin and α-cobratoxin cause an irreversible blockade of the α7AChR-mediated currents in the hippocampus [69]. This interaction is robust and even washing the primary neurons with a toxin-free physiological solution for several hours does not result in any recovery of the ACh-dependent responses, thus pointing to an extremely stable block by these toxins. Our results indicate that mToxin (in nanomolar concentrations) only modulates α7AChR activity and possesses weak and reversible inhibitory properties.

The electrophysiological activation and inactivation of PC-12 cells, shown in Fig. 2, suggest that mToxin significantly decreased the activation and decay periods of nAChR, and partially reduced channel pore opening and closing. Indeed, the reduced motion was predicted theoretically, in our earlier paper with Prof. Michael Levitt, the 2013 Nobel prize laureate in chemistry [37]. In that paper, we calculated normal modes of nAChR and its complex with snake alpha-neurotoxins and found a 30% reduction of twisting motion. This finding was surprising, as snake toxins were thought principally to occlude the acetylcholine binding site, and prevent acetylcholine binding [27]. Here, we showed elegantly that mToxin, which did not occlude the acetylcholine binding site (i.e., ARR1779 still binds and opens the receptor), was still capable of partially immobilizing the receptor twist motion through complex formation, thus confirming our earlier prediction.

In light of the observed effects of mToxin on α7AChR, which are situated pre- and postsynaptically (Fig. 8a), we designed a set of original experiments to examine the compound in brain slices in the context of an original ex vivo model of AD and tested its effects upon numerous synaptic functions. Recent studies indicated the unique effects of α7AChR inhibitors upon receptor activity and memory acquisition. Goethem et al. (2019) elegantly demonstrated that α7AChR antagonists potentiate receptor functioning, increase glutamate efflux, and enhance hippocampal LTP [51]. Remarkably, a low dose of selective α7AChR antagonist methyllycaconitine improves cognitive function in rats [70].

In the present study, we first tested a simple form of presynaptic plasticity by measuring PPF at various interstimulus intervals. This phenomenon is well-studied and related to a transient increase in the vesicular release probability [71]. Of note, PPF ratio values are substantially reduced in transgenic rodent models of AD [72]. Moreover, PPF is diminished in the aged wild-type mice compared to young animals. Lenart et al. reported the PPF ratio at a 50 ms interval of about 1.5 in the six-month-old WT mice [73], which accords with our data (Fig. 3c). In our assay, the pretreatment with Aβ affected this index; however, the effect did not reach statistical significance (Fig. 3d). Of note, the group with mToxin treatment demonstrated the greatest PPF ratios. Thus, we speculate that mToxin influences the levels of intracellular calcium (Fig. 8b), which, in turn, increases the vesicular release probability, even though the mechanism is of this elevation is uncertain. One recent study by He et al. (2019) demonstrated that nanomolar concentrations of Aβ suppress the release probability at the synapses in wild-type mice via depletion of phosphatidylinositol-4,5-bisphosphate in axons (Fig. 8a) [74]. Therefore, we suggest that the phospholipase C pathway might be an interesting target for future investigations of mToxin activity.

To further assess electrophysiological changes associated with Aβ and mToxin treatment, we measured hippocampal LTP, which is the primary experimental model for investigating the synaptic basis of learning and memory [75]. We confirmed the typical Aβ inhibiting effect upon LTP; though, mToxin treatment rescued the Aβ-induced LTP deficits. This effect was very significant in both the initial phase and the maintenance phase of LTP (Fig. 3a). Our results strongly suggest that treatment with mToxin rescues the synaptic transmission deficit caused by Aβ in the LTP paradigm, which models long-term synaptic plasticity and underlies the fundamental mechanisms of learning and memory in mammals [75]. Besides, mToxin significantly improves PPF, which indicates the effects of mToxin upon short-term synaptic plasticity. The mechanisms underlying this phenomenon are exclusively presynaptic [76]. Therefore, mToxin acts via complex pre- and postsynaptic mechanisms (Fig. 8b).

Most noteworthy, mToxin shows no toxicity in brain slices and mice. Its systemic application did not lead to significant changes in mice locomotive behavior (Fig. 4). Consequently, we designed an in vivo experiment to test the effects of mToxin in a rodent model of AD. Since α-neurotoxins do not pass the BBB, we bypassed the barrier and delivered the substance directly into the ventricles using osmotic minipumps. This methodology of chronic slow release ensured a constant concentration of the compound in the brain for about a month. In parallel, wild-type animals underwent the same procedure and tested in the same behavioral setting. We evidenced significant improvement in short-term spatial memory following the treatment with mToxin in AD mice; however, no effect was observed in the wild-type group. Other paradigms did not reveal any effect of the treatment upon memory acquisition and recall in AD mice and as well as in wild-type animals. In this context, we admit that a relatively small number of animals participating in the experiments and a complicated surgery procedure they underwent, increased variability and substantially limited the success of our behavioral study.

Advanced proteomics assay, combined with bioinformatics analysis, served to decipher the molecular mechanisms responsible for the observed phenotype. Previously, another group applied a microarray assay to demonstrate a significant down-regulation of MAPK and ERK1/2 in AD brain tissue compared to control autopsies [77]. Very recently, Zhao et al. (2020) confirmed that the expression levels of pERK1/2 are significantly reduced in the brains of 3×Tg mice compared to wild-type animals [53]. The authors demonstrated that treatment with artemisinin stimulated the phosphorylation of ERK1/2 and subsequent activation of the MAPK/ERK signaling pathway, which eventuated in memory improvement and reduction of apoptosis in this model of AD. Other reports support the hypothesis on a definite role of ERK1/2 in formation, retrieval, reconsolidation, and persistence of memory [78].

We evidenced, by two assays, a significant increase in the levels of ERK1/2 and pERK following the treatment with mToxin. Also, we prove that the activation of ERK is followed by a reduction in the apoptosis rate. In light of recent findings showing a clear correlation between the memory function, brain apoptosis, and levels of pERK in 3×Tg mice, we speculate that this kinase might be responsible, at least partially, for the phenotype observed in our study. In fact, other groups have confirmed a serious involvement of ERK1/2 in α7AChR-mediated and nicotine-dependent downstream signaling [79]. Therefore, this hypothesis is predicated upon strong empirical evidence.

Additionally, we analyzed the hippocampal levels of the toxic oligomeric amyloid species in relation to the treatment with mToxin. We revealed a substantial reduction in the levels of the toxic dodecamers following the treatment, even though the amyloid plaques density did not change significantly (Fig. 6g, i). Of note, activation of α7AChR affects APP processing by regulating secretase activity. Nie et al. (2010) evidenced a significant reduction in Aβ production following nicotine and a specific α7AChR agonist application in vitro [80]. Therefore, improvement in cholinergic signaling due to mToxin effects could lessen the amounts of amyloid oligomers. Moreover, the APP transcription rate is regulated by various cytokines, including tumor necrosis factor, which is secreted by activated microglia and astrocytes. Our proteomics results point to a reduction in astrogliosis and neuroinflammation. Then, we speculate that this mechanism is also possible (Fig. 8b). The extracellular plaque formation is a gradually progressive process, which lasts for decades in men and several months in rodent models. Therefore, we suggest that our experimental design with a relatively short treatment and subsequent cross-sectional analysis is insufficient to induce detectible alterations in the plaque density.

In conclusion, although our experimental design and setting are not optimal, the present study substantially improves the current knowledge of α7AChR function in the brain. Moreover, it explicates the functional consequences of AD-associated, Aβ-driven cholinergic malfunction from molecular to cellular and cognitive levels. Here, we emphasize the complexity of AD pathogenesis together with its extremely uncertain etiology and indeterminate onset, which, in our opinion, are the most significant impediments to finding a competent disease-modifying medicine. Accordingly, we suggest a novel direction in the AD treatment strategy with an alternative and original drug design. Apparently, mToxin cannot be a therapeutic agent due to its poor brain bioavailability. Though, its short but still functional derivatives conjugated with an efficient carrier could represent promising modulators of α7AChR function in the brain that are capable of interfering with the core AD-related pathogenic processes.

## Supplementary Information

Figure S1 The mass spectrum of the modified CTX A characteristic peak at 8397 Da corresponding to the expected mass of the fully modified CTX (7821 Da) in which five arginine residues are substituted with phenylglyoxal (C_8_H_6_O_2_, 134.2 Da × 5 = 671 Da), following the release of five molecules of waters (H_2_O, 18 Da ×5 = 90 Da), and 5 protons (H^+^, 1 Da × 5 = 5 Da). The mass spectrum indicates the complete and successful modification of CTX into mToxin. (PNG 1750 kb)

High resolution image (TIF 1036 kb)

Figure S2 Hippocampal LTP in the CA1 area (female brain slices). Soluble aged Aβ inhibited LTP (red tracing) induced by high-frequency stimulation (HFS, arrow), and was restored by mToxin (yellow tracing, *n* = 9) to its control level (blue tracing, n = 9). mToxin alone had no significant effect on LTP (gray tracing, *n*=9). (PNG 1750 kb)

High resolution image (TIF 879 kb)

Table S1 The list of chemicals and equipment. (DOCX 15 kb)

Table S2 Selected results of the antibody array. CFC - change from control (%). (DOCX 22 kb)

## Data Availability

All data and materials as well as software application or custom code support our claims and comply with field standards. The data that support the findings of this study are available from the corresponding author upon reasonable request.

## Abbreviations

AChR: Nicotinic acetylcholine receptor
αAChR: Nicotinic acetylcholine receptor α-subunit
ACh: Acetylcholine
ACSF: Artificial cerebral spine fluid
AD: Alzheimer’s disease
Aβ: Amyloid-beta
APP: Amyloid precursor protein
BBB: Blood-brain barrier
CFC: Changed from control
CTX: α-cobratoxin
ESI: Electrospray ionization
EPSP: Excitatory postsynaptic potential
FDA: Food and drug administration
fEPSP: Field excitatory postsynaptic potentials
FPLC: Fast protein liquid chromatography
ISI: Interstimulus interval
LTP: Long-term potentiation
MALDI: Matrix-assisted laser desorption/ionization
MWM: Morris water maze
mToxin: Modified snake α-cobratoxin
PDB: Protein Data Bank
PPF: Paired pulse facilitation
PFC: Prefrontal cortex

## References

[1] Selkoe DJ, Hardy J (2016). The amyloid hypothesis of Alzheimer’s disease at 25 years. EMBO Mol Med.

[2] Bartus RT, Dean RL, Beer B, Lippa AS (1982) The cholinergic hypothesis of geriatric memory dysfunction. Science (80-. ) 10.1126/science.7046051 7046051

[3] Contestabile A (2011) The history of the cholinergic hypothesis. Behav Brain Res:334–340 10.1016/j.bbr.2009.12.044 20060018

[4] Bartus RT (1979) Physostigmine and recent memory: Effects in young and aged nonhuman primates. Science (80- ) 10.1126/science.227061 227061

[5] Graham WV, Bonito-Oliva A, Sakmar TP (2017). Update on Alzheimer’s disease therapy and prevention strategies. Annu Rev Med.

[6] Kandimalla R, Reddy PH (2017). Therapeutics of neurotransmitters in Alzheimer’s disease. J Alzheimers Dis.

[7] Glenner GG, Wong CW (1984). Alzheimer’s disease: initial report of the purification and characterization of a novel cerebrovascular amyloid protein. Biochem Biophys Res Commun.

[8] Hardy J, Allsop D (1991). Amyloid deposition as the central event in the aetiology of Alzheimer’s disease. Trends Pharmacol Sci.

[9] Polis B, Samson A (2019) A new perspective on Alzheimer’s disease as a brain expression of a complex metabolic disorder. In: Wisniewski T (ed) Alzheimer’s Dis. 1st ed, Brisbane, pp. 1–22 31895518

[10] Lam B, Masellis M, Freedman M, Stuss DT, Black SE (2013) Clinical, imaging, and pathological heterogeneity of the Alzheimer’s disease syndrome. Alzheimer’s Res Ther 10.1186/alzrt155 PMC3580331 23302773

[11] Lendvai B, Kassai F (2013). Szájli ágota, Némethy Z. α7 Nicotinic acetylcholine receptors and their role in cognition. Brain Res Bull.

[12] Dineley KT, Pandya AA, Yakel JL (2015). Nicotinic ACh receptors as therapeutic targets in CNS disorders. Trends Pharmacol Sci.

[13] Dani JA, Bertrand D (2007). Nicotinic acetylcholine receptors and nicotinic cholinergic mechanisms of the central nervous system. Annu Rev Pharmacol Toxicol.

[14] Samson AO, Chill JH, Rodriguez E, Scherf T, Anglister J (2001). NMR mapping and secondary structure determination of the major acetylcholine receptor alpha-subunit determinant interacting with alpha-bungarotoxin. Biochemistry..

[15] Wonnacott S. Nicotinic ACh Receptors. Tocris Sci Rev Ser. 2014;

[16] Kabbani N, Nordman JC, Corgiat BA, Veltri DP, Shehu A, Seymour VA (2013). Are nicotinic acetylcholine receptors coupled to G proteins? BioEssays.

[17] Parri HR, Hernandez CM, Dineley KT (2011). Research update: Alpha7 nicotinic acetylcholine receptor mechanisms in Alzheimer’s disease. Biochem Pharmacol.

[18] De Strooper B (2010). Proteases and proteolysis in Alzheimer disease: a multifactorial view on the disease process. Physiol Rev.

[19] Sakono M, Zako T (2010). Amyloid oligomers: formation and toxicity of Aβ oligomers. FEBS J.

[20] Smith LM, Strittmatter SM (2017) Binding sites for amyloid-β oligomers and synaptic toxicity. Cold Spring Harb Perspect Med 10.1101/cshperspect.a024075 PMC5411685 27940601

[21] Wang HY, Lee DHS, Davis CB, Shank RP (2000). Amyloid peptide Aβ1-42 binds selectively and with picomolar affinity to α7 nicotinic acetylcholine receptors. J Neurochem.

[22] Liu Q, Kawai H, Berg DK (2001). Beta -amyloid peptide blocks the response of alpha 7-containing nicotinic receptors on hippocampal neurons. Proc Natl Acad Sci U S A.

[23] Nagele RG, D’Andrea MR, Anderson WJ, Wang H-Y (2002). Intracellular accumulation of beta-amyloid(1-42) in neurons is facilitated by the alpha 7 nicotinic acetylcholine receptor in Alzheimer’s disease. Neuroscience..

[24] Lasala M, Fabiani C, Corradi J, Antollini S, Bouzat C, Sadigh-eteghad S (2019). Molecular modulation of human α7 nicotinic receptor by amyloid-β peptides. Front Cell Neurosci [Internet]. Tehran Univ Med Sci.

[25] Bouzat C, Mukhtasimova N (2018) The nicotinic acetylcholine receptor as a molecular machine for neuromuscular transmission. Curr Opin Physiol

[26] Dineley KT, Bell KA, Bui D, Sweatt JD (2002). β-Amyloid peptide activates α7 nicotinic acetylcholine receptors expressed in Xenopus oocytes. J Biol Chem.

[27] Samson A, Scherf T, Eisenstein M, Chill J, Anglister J (2002). The mechanism for acetylcholine receptor inhibition by alpha-neurotoxins and species-specific resistance to alpha-bungarotoxin revealed by NMR. Neuron..

[28] Maatuk N, Samson AO (2013). Modeling the binding mechanism of Alzheimer’s Aβ1-42 to nicotinic acetylcholine receptors based on similarity with snake α-neurotoxins. Neurotoxicology..

[29] Walker JM (2002) The protein protocols handbook. Humana Press

[30] Takahashi K (1968) The reaction of phenylglyoxal with arginine residues in proteins. J Biol Chem 5723461

[31] Yang CC, Chang CC, Liou IF (1974). Studies on the status of arginine residues in cobrotoxin. Biochim Biophys Acta - Protein Struct.

[32] Schrödinger L (2015) The PyMol Molecular Graphics System, Versión 1.8. Thomas Hold

[33] Duhovny D, Nussinov R, Wolfson HJ (2002). Efficient unbound docking of rigid molecules. Lect Notes Comput Sci (including Subser Lect Notes Artif Intell Lect Notes Bioinformatics).

[34] Schneidman-Duhovny D, Inbar Y, Nussinov R, Wolfson HJ (2005) PatchDock and SymmDock: Servers for rigid and symmetric docking. Nucleic Acids Res. 10.1093/nar/gki481 PMC1160241 15980490

[35] Naldi M, Fiori J, Pistolozzi M, Drake AF, Bertucci C, Wu R, Mlynarczyk K, Filipek S, de Simone A, Andrisano V (2012). Amyloid β-peptide 25-35 self-assembly and its inhibition: a model undecapeptide system to gain atomistic and secondary structure details of the Alzheimers disease process and treatment. ACS Chem Neurosci.

[36] Fonar G, Polis B, Meirson T, Maltsev A, Elliott E, Samson AO (2018) Intracerebroventricular administration of L-arginine improves spatial memory acquisition in triple transgenic mice via reduction of oxidative stress and apoptosis. Transl Neurosci 10.1515/tnsci-2018-0009 PMC5984558 29876138

[37] Samson AO, Levitt M (2008) Inhibition mechanism of the acetylcholine receptor by α-neurotoxins as revealed by normal-mode dynamics. Biochemistry 10.1021/bi702272j PMC2750825 18327915

[38] Alama A, Bruzzo C, Cavalieri Z, Forlani A, Utkin Y, Casciano I et al (2011) Inhibition of the nicotinic acetylcholine receptors by cobra venom α-neurotoxins: Is there a perspective in lung cancer treatment? PLoS One 10.1371/journal.pone.0020695 PMC3113800 21695184

[39] Oddo S, Caccamo A, Shepherd JD, Murphy MP, Golde TE, Kayed R, Metherate R, Mattson MP, Akbari Y, LaFerla FM (2003). Triple-transgenic model of Alzheimer’s disease with plaques and tangles: intracellular Aβ and synaptic dysfunction. Neuron..

[40] Fonar G, Polis B, Meirson T, Maltsev A, Samson AO (2018). Subcutaneous sustained-release of poly-arginine ameliorates cognitive impairment in a transgenic mouse model of Alzheimer’ s disease. Adv Alzheimer’s Dis.

[41] Polis B, Srikanth KD, Elliott E, Gil-Henn H, Samson AO (2018) L-norvaline reverses cognitive decline and synaptic loss in a murine model of Alzheimer’s disease. Neurotherapeutics 10.1007/s13311-018-0669-5 PMC6277292 30288668

[42] Polis B, Srikanth K, Gurevich V, Gil-Henn H, Samson A (2019). L-Norvaline, a new therapeutic agent against Alzheimer’s disease. Neural Regen Res.

[43] Kastritis PL, Bonvin AMJJ (2013). On the binding affinity of macromolecular interactions: Daring to ask why proteins interact. J R Soc Interface.

[44] Chang KT, Berg DK (1999). Nicotinic acetylcholine receptors containing α7 subunits are required for reliable synaptic transmission in situ. J Neurosci.

[45] Crowley LC, Marfell BJ, Waterhouse NJ (2016) Detection of DNA fragmentation in apoptotic cells by TUNEL. Cold Spring Harb Protoc 10.1101/pdb.prot087221 27698233

[46] Gadad BS, Britton GB, Rao KS (2011). Targeting oligomers in neurodegenerative disorders: lessons from α-synuclein, tau, and amyloid-β peptide. J Alzheimers Dis.

[47] Gao F, Chen D, Ma X, Sudweeks S, Yorgason JT, Gao M, Turner D, Eaton JB, McIntosh JM, Lukas RJ, Whiteaker P, Chang Y, Steffensen SC, Wu J (2019). Alpha6-containing nicotinic acetylcholine receptor is a highly sensitive target of alcohol. Neuropharmacology..

[48] Mucke L, Selkoe DJ (2012) Neurotoxicity of amyloid β-protein: synaptic and network dysfunction. Cold Spring Harb Perspect Med 2 10.1101/cshperspect.a006338 PMC3385944 22762015

[49] Schmid AW, Lynch MA, Herron CE (2009). The effects of IL-1 receptor antagonist on beta amyloid mediated depression of LTP in the rat CA1 in vivo. Hippocampus..

[50] Frey U, Huang YY, Kandel ER. Effects of cAMP simulate a late stage of LTP in hippocampal CA1 neurons. Science (80- ). 1993; 10.1126/science.8389057 8389057

[51] van Goethem NP, Paes D, Puzzo D, Fedele E, Rebosio C, Gulisano W et al (2019) Antagonizing α7 nicotinic receptors with methyllycaconitine (MLA) potentiates receptor activity and memory acquisition. Cell Signal 10.1016/j.cellsig.2019.06.003 31176021

[52] Dickson DW (2004) Apoptotic mechanisms in Alzheimer neurofibrillary degeneration: Cause or effect? J Clin Invest:23–27 10.1172/JCI22317 PMC437977 15232608

[53] Zhao X, Li S, Gaur U, Zheng W (2020). Artemisinin improved neuronal functions in Alzheimer’s disease animal model 3xtg mice and neuronal cells via stimulating the ERK/CREB signaling pathway. Aging Dis.

[54] Jährling N, Becker K, Wegenast-Braun BM, Grathwohl SA, Jucker M, Dodt HU (2015) Cerebral β-amyloidosis in mice investigated by ultramicroscopy. PLoS One 10.1371/journal.pone.0125418 PMC4446269 26017149

[55] Young KF, Pasternak SH, Rylett RJ (2009). Oligomeric aggregates of amyloid β peptide 1-42 activate ERK/MAPK in SH-SY5Y cells via the α7 nicotinic receptor. Neurochem Int.

[56] Maguschak KA, Ressler KJ (2008). β-Catenin is required for memory consolidation. Nat Neurosci.

[57] Kelleher RJ, Govindarajan A, Jung HY, Kang H, Tonegawa S (2004) Translational control by MAPK signaling in long-term synaptic plasticity and memory. Cell 10.1016/s0092-8674(04)00115-1 15016380

[58] Chen LL, Wang YB, Song JX, Deng WK, Lu JH, Ma LL, Yang CB, Li M, Xue Y (2017). Phosphoproteome-based kinase activity profiling reveals the critical role of MAP2K2 and PLK1 in neuronal autophagy. Autophagy..

[59] Korolainen MA, Auriola S, Nyman TA, Alafuzoff I, Pirttilä T (2005) Proteomic analysis of glial fibrillary acidic protein in Alzheimer’s disease and aging brain. Neurobiol Dis. 10.1016/j.nbd.2005.05.021 15979880

[60] Osborn LM, Kamphuis W, Wadman WJ, Hol EM (2016) Astrogliosis: An integral player in the pathogenesis of Alzheimer’s disease. Prog Neurobiol:121–141 10.1016/j.pneurobio.2016.01.001 26797041

[61] Senechal Y, Larmet Y, Dev KK (2006) Unraveling in vivo functions of amyloid precursor protein: Insights from knockout and knockdown studies. Neurodegener Dis 10.1159/000094772 16954700

[62] Garcia-Osta A, Alberini CM (2009) Amyloid beta mediates memory formation. Learn Mem. 10.1101/lm.1310209 PMC2661754 19318468

[63] Dineley KT (2007). Beta-amyloid peptide - Nicotinic acetylcholine receptor interaction: The two faces of health and disease. Front Biosci.

[64] Puzzo D, Gulisano W, Arancio O, Palmeri A (2015) The keystone of Alzheimer pathogenesis might be sought in Aβ physiology. Neuroscience 10.1016/j.neuroscience.2015.08.039 PMC4591241 26314631

[65] Buckingham SD, Jones AK, Brown LA, Sattelle DB (2009). Nicotinic acetylcholine receptor signalling: roles in Alzheimer’s disease and amyloid neuroprotection. Pharmacol Rev.

[66] Hu M, Waring JF, Gopalakrishnan M, Li J (2008) Role of GSK-3β activation and α7 nAChRs in Aβ 1–42-induced tau phosphorylation in PC12 cells. J Neurochem.:1371–1377 10.1111/j.1471-4159.2008.05483.x 18485099

[67] Lawrence JLM, Tong M, Alfulaij N, Sherrin T, Contarino M, White MM, Bellinger FP, Todorovic C, Nichols RA (2014). Regulation of presynaptic Ca2+, synaptic plasticity and contextual fear conditioning by a N-terminal β-amyloid fragment. J Neurosci.

[68] Del Brutto OH, Del Brutto VJ (2012) Neurological complications of venomous snake bites: a review. Acta Neurol Scand:363–372 10.1111/j.1600-0404.2011.01593.x 21999367

[69] Alkondon M, Albuquerque EX (1991) Initial characterization of the nicotinic acetylcholine receptors in rat hippocampal neurons. J Recept Signal Transduct 10.3109/10799899109064693 1753378

[70] Hahn B, Shoaib M, Stolerman IP (2011). Selective nicotinic receptor antagonists: Effects on attention and nicotine-induced attentional enhancement. Psychopharmacology.

[71] Satake S, Inoue T, Imoto K (2012). Paired-pulse facilitation of multivesicular release and intersynaptic spillover of glutamate at rat cerebellar granule cell-interneurone synapses. J Physiol.

[72] Tomiyama T, Matsuyama S, Iso H, Umeda T, Takuma H, Ohnishi K, Ishibashi K, Teraoka R, Sakama N, Yamashita T, Nishitsuji K, Ito K, Shimada H, Lambert MP, Klein WL, Mori H (2010). A mouse model of amyloid β oligomers: their contribution to synaptic alteration, abnormal tau phosphorylation, glial activation, and neuronal loss in vivo. J Neurosci.

[73] Lénárt N, Szegedi V, Juhász G, Kasztner A, Horváth J, Bereczki E, Tóth ME, Penke B, Sántha M (2012). Increased tau phosphorylation and impaired presynaptic function in hypertriglyceridemic ApoB-100 transgenic mice. PLoS One.

[74] He Y, Wei M, Wu Y, Qin H, Li W, Ma X et al (2019) Amyloid β oligomers suppress excitatory transmitter release via presynaptic depletion of phosphatidylinositol-4,5-bisphosphate. Nat Commun 10.1038/s41467-019-09114-z PMC6416269 30867420

[75] Bliss TVP, Collingridge GL (1993). A synaptic model of memory: long-term potentiation in the hippocampus. Nature..

[76] Zucker RS, Regehr WG (2002). Short-term synaptic plasticity. Annu Rev Physiol.

[77] Loring JF, Wen X, Lee JM, Seilhamer J, Somogyi R (2001) A gene expression profile of Alzheimer’s disease. DNA Cell Biol 10.1089/10445490152717541 11788046

[78] Medina JH, Viola H (2018). ERK1/2: A key cellular component for the formation, retrieval, reconsolidation and persistence of memory. Front Mol Neurosci.

[79] Shi D, Guo W, Chen W, Fu L, Wang J, Tian Y et al (2012) Nicotine promotes proliferation of human nasopharyngeal carcinoma cells by regulating α7AChR, ERK, HIF-1α and VEGF/PEDF signaling. PLoS One 10.1371/journal.pone.0043898 PMC3432052 22952803

[80] Nie HZ, Shi S, Lukas RJ, Zhao WJ, Sun YN, Yin M (2010). Activation of α7 nicotinic receptor affects APP processing by regulating secretase activity in SH-EP1-α7 nAChR-hAPP695 cells. Brain Res.

