# Supplementary figures and images for: Modified Snake α-Neurotoxin Averts β-Amyloid Binding to α7 Nicotinic Acetylcholine Receptor and Reverses Cognitive Deficits in Alzheimer’s Disease Mice
Source: Mol Neurobiol. 2021 Jan 8;58(5):2322–41. doi: 10.1007/s12035-020-02270-0 (PMC8018932; doi:10.1007/s12035-020-02270-0)

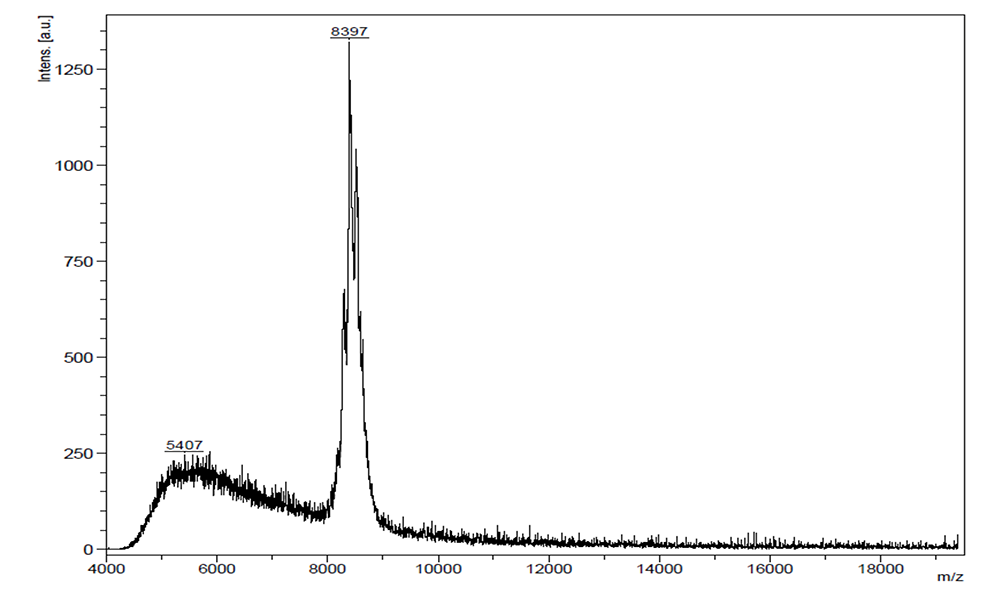

Supplement: Supplementary file 1 — The mass spectrum of the modified CTX A characteristic peak at 8397 Da corresponding to the expected mass of the fully modified CTX (7821 Da) in which five arginine residues are substituted with phenylglyoxal (C8H6O2, 134.2 Da × 5 = 671 Da), following the release of five molecules of waters (H2O, 18 Da ×5 = 90 Da), and 5 protons (H+, 1 Da × 5 = 5 Da). The mass spectrum indicates the complete and successful modification of CTX into mToxin. (PNG 1750 kb) [file 12035_2020_2270_Fig9_ESM.png]

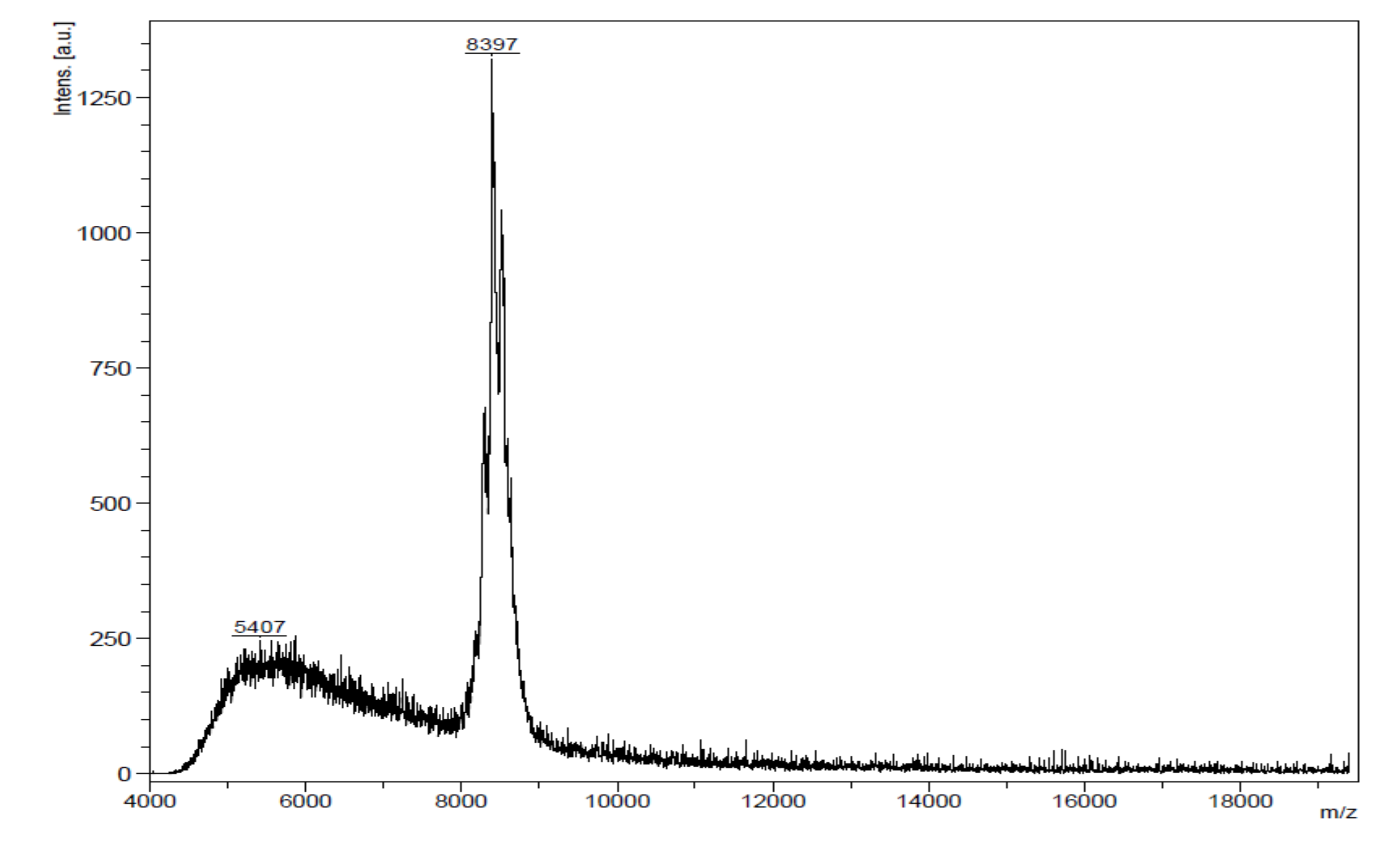

Supplement: Supplementary file 2 — High resolution image (TIF 1036 kb) [file 12035_2020_2270_MOESM1_ESM.tif]

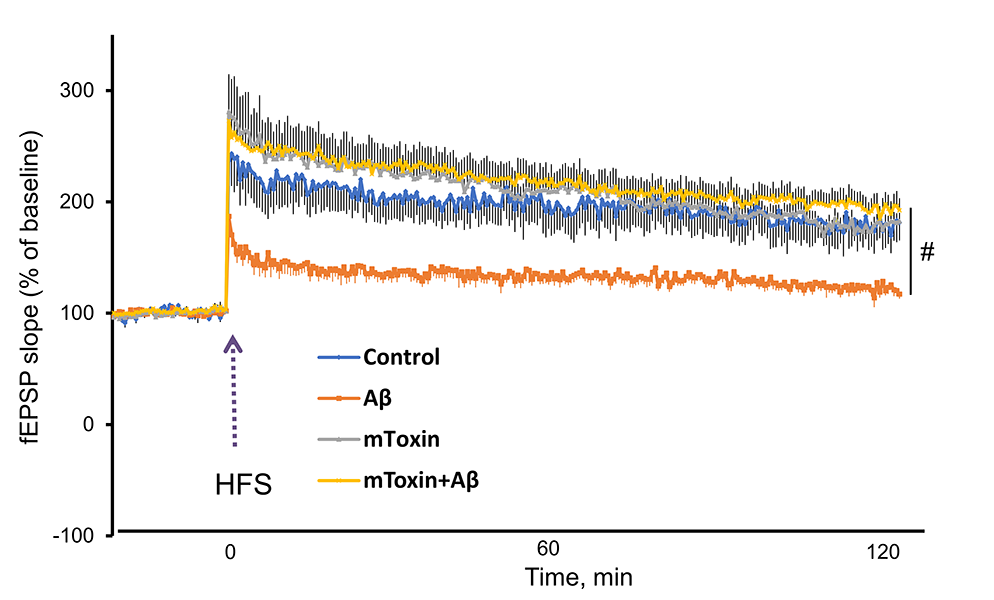

Supplement: Supplementary file 3 — Hippocampal LTP in the CA1 area (female brain slices). Soluble aged Aβ inhibited LTP (red tracing) induced by high-frequency stimulation (HFS, arrow), and was restored by mToxin (yellow tracing, n = 9) to its control level (blue tracing, n = 9). mToxin alone had no significant effect on LTP (gray tracing, n=9). (PNG 1750 kb) [file 12035_2020_2270_Fig10_ESM.png]

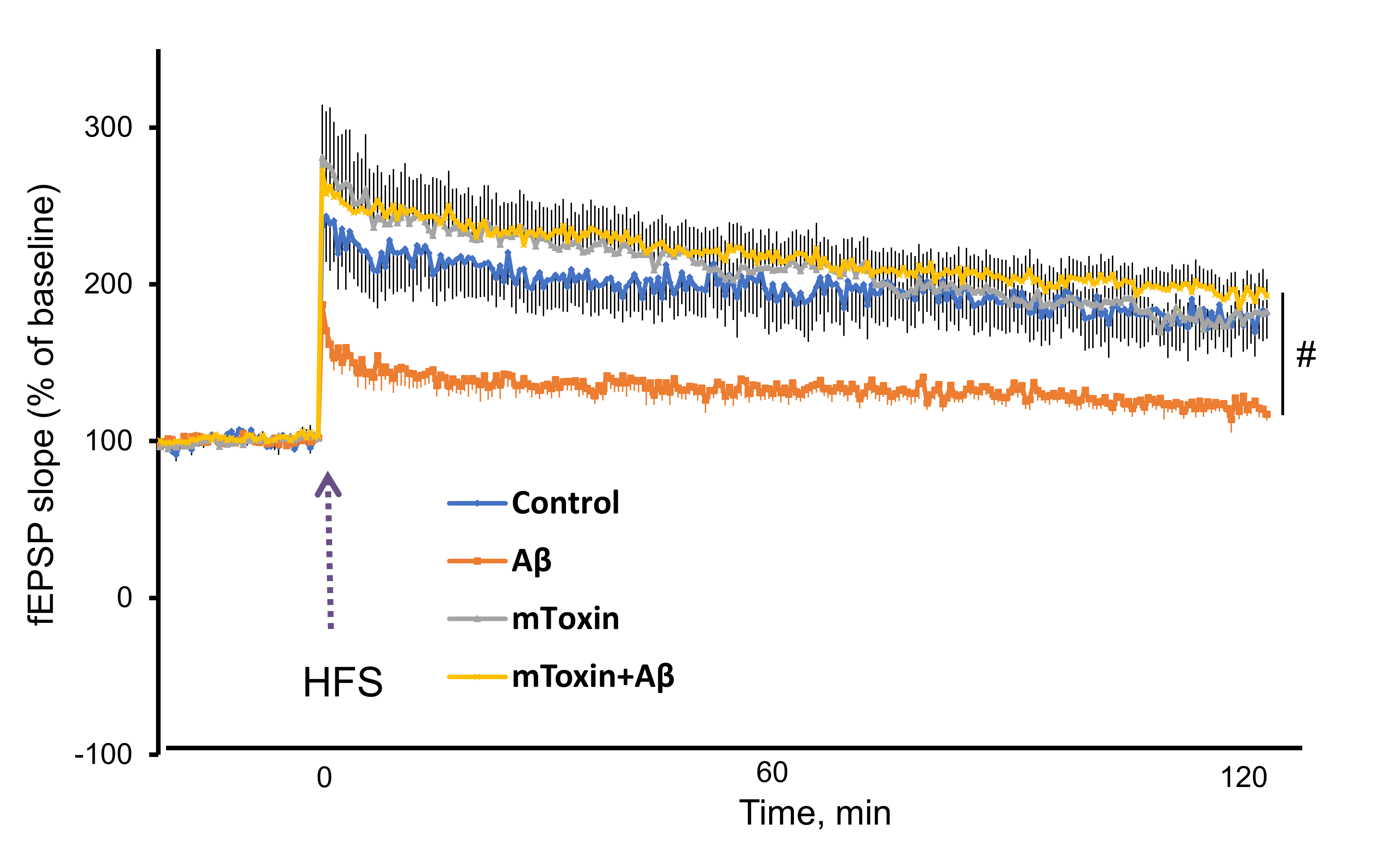

Supplement: Supplementary file 4 — High resolution image (TIF 879 kb) [file 12035_2020_2270_MOESM2_ESM.tif]
