# Supplementary material for: Modified Snake α-Neurotoxin Averts β-Amyloid Binding to α7 Nicotinic Acetylcholine Receptor and Reverses Cognitive Deficits in Alzheimer’s Disease Mice
Source: Mol Neurobiol. 2021 Jan 8;58(5):2322–41. doi: 10.1007/s12035-020-02270-0 (PMC8018932; doi:10.1007/s12035-020-02270-0)
Supplement: Supplementary file 5 — The list of chemicals and equipment. (DOCX 15 kb) [file 12035_2020_2270_MOESM3_ESM.docx]

# Supplementary table 1. Chemicals and Equipment.

| **Reagents** | **Supplier** | **Catalogue #** | **Lot#** |
| --- | --- | --- | --- |
| 1640 medium | Corning | 10040-CVR | 04216004 |
| Fetal Bovine Serum (FBS) | Gibco | 10270-106 | 42Q7760K |
| horse serum | Solarbio | S9050 | 1110B051 |
| 0.25%-Trypsin-EDTA | Gibco | 25200-072 | 1868709 |
| EGTA | Sigma | E3889 | SLBP2806V |
| HEPES | Santa Cruz | SC-29097A | C2817 |
| MgCl2 | Sigma | M2393 | SLBT5995 |
| KCl | Sigma | P5405 | SLBR2609V |
| NaCl | Sigma | S5886 | SLBT7002 |
| Glucose | Sigma | G8270 | SLBV7620 |
| CaCl2 | Sigma | C7902 | SLBV3136 |
| NaH2PO4 | General Reagent | G21298B | P1297763 |
| CsCl | Amresco | 0415-50G | 1614C118 |
| CsF | Innochem | A44779 | KSBEU26 |
| PBS | Takara | T900 | 2301 |

| Equipment | **Vendor** | **Model** |
| --- | --- | --- |
| Amplifier | HEKA (Germany) | EPC10 |
| Micro-manupulator | MCI Instruments | 86PW420600 |
| Micropipette puller | Sutter Instruments (USA) | P97 |
| Microscope | Motic | AE31E |
| Glass pipette | Sutter Instruments (USA) | BF150-86-10 |
| Software for data acquisition | HEKA (Germany) | Patchmaster &IGOR Pro |
