# Supplementary material for: Modified Snake α-Neurotoxin Averts β-Amyloid Binding to α7 Nicotinic Acetylcholine Receptor and Reverses Cognitive Deficits in Alzheimer’s Disease Mice
Source: Mol Neurobiol. 2021 Jan 8;58(5):2322–41. doi: 10.1007/s12035-020-02270-0 (PMC8018932; doi:10.1007/s12035-020-02270-0)
Supplement: Supplementary file 6 — Selected results of the antibody array. CFC - change from control (%). (DOCX 22 kb) [file 12035_2020_2270_MOESM4_ESM.docx]

| **Full Target Protein Name** | **Phospho Site** | **%CFC** |
| --- | --- | --- |
| STE20-like serine/threonine-protein kinase (STK2) | S189 | 452 |
| Catenin (cadherin-associated protein) beta 1 (CTNNB1) | Pan-specific | 434 |
| B23 (nucleophosmin, numatrin, nucleolar protein NO38) | T199 | 417 |
| MAPK/ERK protein-serine kinase 1 (MKK1, MAP2K1) | Pan-specific | 349 |
| Signal transducer and activator of transcription 5B | Pan-specific | 342 |
| RAC-alpha serine/threonine-protein kinase | Y315 | 328 |
| Cyclin-dependent protein-serine kinase 7 | Pan-specific | 324 |
| Gamma-enolase | Y25 | 309 |
| Beta-adrenergic receptor kinase 1 (GRK2, ADRBK1) | S670 | 307 |
| MAPK/ERK protein-serine kinase 4 (MEK4, MAP2K4) | S257 | 290 |
| p21-activated kinase 4 | S474 | 288 |
| Aurora Kinase B (serine/threonine protein kinase 12), (AIM-1) | T232 | 275 |
| Alpha-synuclein | S129 | 272 |
| Protein-serine kinase C theta (PRKCQ) | Pan-specific | 256 |
| Protein-serine kinase C zeta (PRKCZ) | S262+Y263 | 247 |
| Cyclin-dependent protein-serine kinase 2 | T160 | 246 |
| Janus protein-tyrosine kinase 2 | Y1007+Y1008 | 239 |
| Cyclin-dependent protein-serine kinase 1/2 (CDC2) | T14+Y15 | 233 |
| Tyrosine-protein kinase Mer (MER) | Y749 | 230 |
| Protein-tyrosine kinase 2 (Jak-related) | Pan-specific | 228 |
| Cyclin-dependent protein-serine kinase 1/2 (CDC2) | Pan-specific | 220 |
| Aurora Kinase A (serine/threonine protein kinase 6), (AIK, STK15) | T287+T288 | 219 |
| Mammalian STE20-like protein-serine kinase 1 (KRS2, STK4) | Pan-specific | 216 |
| Abelson proto-oncogene-encoded protein-tyrosine kinase | Y139 | 213 |
| Glycogen synthase-serine kinase 3 alpha | Y284+Y285 | 213 |
| Bone marrow X protein-tyrosine kinase (Etk) | Y40 | 211 |
| Calcium/calmodulin-dependent protein-serine kinase 4 (CaMPK4) | Pan-specific | 202 |
| Twinfilin-2 | Y309 | 202 |
| MAPK/ERK protein-serine kinase 4 (MEK4, MAP2K4) | Pan-specific | 199 |
| Protein-serine kinase C lambda/iota (PRKCI) | Pan-specific | 195 |
| Tyrosine-protein kinase ABL2 | Y439 | 193 |
| Ephrin type-A receptor 2 protein-tyrosine kinase | Y772 | 191 |
| Cyclin-dependent protein-serine kinase 1 (CDC2) | Y19 | 190 |
| Ephrin type-B receptor 2 protein-tyrosine kinase | Y780 | 187 |
| RAC-alpha serine/threonine-protein kinase | Y326 | 185 |
| Spleen protein-tyrosine kinase | Y525+Y526 | 182 |
| Focal adhesion protein-tyrosine kinase | Y397 | 178 |
| Extracellular regulated protein-serine kinase 1 (p44 MAP kinase) | Y204 | 178 |
| MAPK/ERK protein-serine kinase 2 (MKK2, MAP2K2) | Pan-specific | 174 |
| Fyn proto-oncogene-encoded protein-tyrosine kinase | Y213+Y214 | 172 |
| Cyclin-dependent protein-serine kinase 9 | T186 | 172 |
| Caveolin 1 (CAV1) | Pan-specific | 171 |
| Focal adhesion protein-tyrosine kinase | Y577 | 170 |
| Extracellular regulated protein-serine kinase 5 (Big MAP kinase 1 (BMK1)) | Y221 | 170 |
| Extracellular regulated protein-serine kinase 4 | Pan-specific | 168 |
| Mitotic checkpoint serine/threonine-protein kinase BUB1 beta (Bub1R) | S670 | 165 |
| Protein-serine kinase C beta 1 (PRKCB1) | T500 | 165 |
| Inhibitor of NF-kappa-B alpha (MAD3) | Pan-specific | 164 |
| ErbB2 (Neu) receptor-tyrosine kinase | Y877 | 161 |
| Ephrin type-A receptor 1 protein-tyrosine kinase | Pan-specific | 156 |
| PCTAIRE-1 protein-serine kinase (CDK16, PCTK1) | Y176 | 155 |
| Protein-serine kinase C alpha (PRKCA) | Pan-specific | 155 |
| Cyclin-dependent protein-serine kinase 5 | Y15 | 154 |
| Calcium/calmodulin-dependent protein-serine kinase 1 alpha (CaMKI) | T177 | 151 |
| Raf1 proto-oncogene-encoded protein-serine kinase (RafC) | S301+T303 | 148 |
| p21-activated kinase 2 (gamma) (serine/threonine-protein kinase PAK 2) (PAKg) | Y130 | 148 |
| NF-kappa-B p50 nuclear transcription factor | Pan-specific | 147 |
| Phosphatidylinositol 3-kinase regulatory subunit beta | Y464 | 142 |
| Aurora Kinase B (serine/threonine protein kinase 12), (AIM-1) | Pan-specific | 142 |
| Actin, cytoplasmic 1 (Beta-actin) | Y53 | 141 |
| Integrin-linked protein-serine kinase 1 | Y351 | 141 |
| Extracellular regulated protein-serine kinase 1 (p44 MAP kinase) | Y204+T207 | 141 |
| Receptor tyrosine-protein kinase erbB-4 | Y733 | 141 |
| Calcium/calmodulin-dependent protein-serine kinase 4 (CaMPK4) | T200 | 139 |
| Hepatocyte growth factor (HGF) receptor-tyrosine kinase | Y1234+Y1235+S1236 | 139 |
| MAPK/ERK protein-serine kinase 1 (MKK1, MAP2K1) | S222 | 135 |
| Inhibitor of NF-kappa-B beta (thyroid receptor interacting protein 9) | Pan-specific | 135 |
| Serine/threonine-protein kinase TBK1 | Pan-specific | 128 |
| Protein-serine kinase C delta (PRKCD) | Y334 | 128 |
| Ribosomal S6 protein-serine kinase 1/2 (RPS6KA1/A3, p90RSK) | S363/S369 | 127 |
| Calcium/calmodulin-dependent protein-serine kinase 1 delta | Pan-specific | 125 |
| Cyclin-dependent protein-serine kinase 6 | Y24 | 125 |
| Fibroblast growth factor receptor-tyrosine kinase 2 (BEK) | Y656+Y657 | 124 |
| Mitogen-activated protein-serine kinase p38 alpha (MAPK14) | T180+Y182 | 120 |
| B lymphoid tyrosine kinase | Y389 | 120 |
| Mitogen-activated protein kinase kinase kinase kinase 4 (ZC1, MAP4K4) | T187 | 117 |
| Tyrosine-protein kinase receptor UFO | Y702+Y703 | 117 |
| Ataxia telangiectasia mutated | S1981 | 117 |
| Microtubule-associated protein tau | S739 | 115 |
| RAC-alpha serine/threonine-protein kinase | T308 | 114 |
| Gardner-Rasheed feline sarcoma viral (v-fgr) oncogene | Y412 | 114 |
| MAPK/ERK protein-serine kinase 4 (MEK4, MAP2K4) | S80 | 113 |
| Aurora Kinase B (serine/threonine protein kinase 12), (AIM-1) | Pan-specific | 112 |
| Beta-adrenergic receptor kinase 1 (GRK2, ADRBK1) | Y356 | 112 |
| Apoptosis signal regulating protein-serine kinase 1 (MAP3K5) | S1033 | 111 |
| Inhibitor of nuclear factor kappa-B kinase subunit epsilon (IkBKE) | S172 | 111 |
| Activating transcription factor 2 (CRE-BP1) | S112 | 110 |
| Protein-tyrosine kinase 6 (PTK6) | Y342 | 110 |
| Enhancer of filamentation 1 | Y166 | 109 |
| Tyrosine-protein kinase Mer (MER) | Y753 | 109 |
| Wee1 protein-tyrosine kinase | Pan-specific | 109 |
| Cell division protein kinase 17 (CDK17, PCTK2) | S180 | 109 |
| Insulin-like growth factor 1 receptor protein-tyrosine kinase | Y1346 | 108 |
| Cyclin-dependent protein-serine kinase 1 (CDC2) | Pan-specific | 108 |
| Inhibitor of NF-kappa-B protein-serine kinase alpha (CHUK, IkBKA) | Pan-specific | 108 |
| Cyclin-dependent protein-serine kinase 6 | Y13 | 108 |
| p21-activated kinase 2 (gamma) (serine/threonine-protein kinase PAK 2) (PAKg) | S141 | 107 |
| Apoptosis signal regulating protein-serine kinase 1 (MAP3K5) | Pan-specific | 104 |
| Annexin A2 | Y238 | 104 |
| Fer (fps/fes related) tyrosine kinase | Y402 | 104 |
| Abelson proto-oncogene-encoded protein-tyrosine kinase | Y226 | 103 |
| Lymphocyte-oriented protein-serine kinase | T952 | 103 |
| Hepatocyte growth factor (HGF) receptor-tyrosine kinase | T1241 | 103 |
| NF-kappa-B p65 nuclear transcription factor (Rel A) | Pan-specific | 101 |
| Polymerase I and transcript release factor | Y308 | 100 |
| Cyclin-dependent protein-serine kinase 9 | S347 | 100 |
| Ephrin type-B receptor 1 protein-tyrosine kinase | Y594 | 100 |
| Protein-tyrosine kinase 2 (PTK2B) | Y402 | 100 |
| MAPK/ERK kinase kinase 2 (MAP3K2) | S239 | 100 |
| Ataxia telangiectasia mutated | Y2969 | 99 |
| Annexin A1 | Y207 | 99 |
| Signal transducer and activator of transcription 2 | Pan-specific | 98 |
| Cyclin-dependent protein-serine kinase 4 | Pan-specific | 97 |
| Adducin alpha, gamma (ADD 1/3) | S726 | 96 |
| Platelet-derived growth factor receptor kinase alpha | S847+Y849 | 96 |
| Calcium/calmodulin-dependent protein-serine kinase 4 (CaMPK4) | Pan-specific | 95 |
| Protein-tyrosine kinase 6 (PTK6) | S446+Y447 | 95 |
| Extracellular regulated protein-serine kinase 2 (p42 MAP kinase) | Pan-specific | 95 |
| Checkpoint protein-serine kinase 1 (CHEK1) | S345 | 92 |
| Tyrosine-protein kinase receptor TYRO3 | Y681 | 91 |
| Mitogen-activated protein kinase-activated protein kinase 2 | Y225+T226 | 90 |
| Protein-serine kinase C mu (Protein kinase D) (PRKD1, PKD1, PRKCM) | S910 | 89 |
| Fes/Fps protein-tyrosine kinase | Pan-specific | 89 |
| Extracellular regulated protein-serine kinase 1 (p44 MAP kinase) | T202+Y204 | 88 |
| Checkpoint protein-serine kinase 1 (CHEK1) | S317 | 86 |
| Jun N-terminus protein-serine kinase (stress-activated protein kinase (SAPK)) 1 (SAPKg, MAPK8) | Y185 | 86 |
| Extended synaptotagmin-1 | Y822 | 86 |
| Cyclin-dependent protein-serine kinase 2 | Pan-specific | 86 |
| Serine/threonine-protein kinase TAO1 (TAOK1) | S181 | 85 |
| p21-activated kinase 2 (gamma) (serine/threonine-protein kinase PAK 2) (PAKg) | Pan-specific | 85 |
| p21-activated kinase 3 (beta) (serine/threonine-protein kinase PAK 3) (PAKb) | Pan-specific | 84 |
| Osaka thyroid oncogene protein-serine kinase (TPL2) (MAP3K8) | Pan-specific | 83 |
| Glycogen synthase-serine kinase 3 beta | Pan-specific | 83 |
| Tyrosine kinase-type cell surface receptor HER3 | Y1307 | 80 |
| Cyclin-dependent protein-serine kinase 10 (PISSLRE) | T196 | 80 |
| Cyclin-dependent protein-serine kinase 6 | Pan-specific | 80 |
| Vascular endothelial growth factor receptor-tyrosine kinase 2 (Flk1) | Y1214 | 80 |
| Bone morphogenetic protein receptor type-2 | S375 | 80 |
| Macrophage colony-stimulating factor 1 receptor (Fms) | Y699 | 79 |
| Ribosomal protein S6 kinase beta-1 (RPS6KB1 p70S6Ka) | Pan-specific | 78 |
| Twinfilin-1 | Y309 | 78 |
| Huntington's disease protein | S421 | 78 |
| MAPK/ERK protein-serine kinase 1 (MKK1, MAP2K1) | Pan-specific | 77 |
| Focal adhesion protein-tyrosine kinase | Y576+Y577 | 77 |
| Protein-serine kinase C theta (PRKCQ) | S695 | 75 |
| Hepatocyte growth factor (HGF) receptor-tyrosine kinase | S1236 | 74 |
| Vav 1 guanine nucleotide exchange factor | Y826 | 74 |
| Bruton's agammaglobulinemia tyrosine kinase | Y223+Y225 | 74 |
| Fes/Fps protein-tyrosine kinase | Y713+S716 | 73 |
| Protein-serine kinase suppressor of Ras 1 | Pan-specific | 73 |
| MOK protein kinase (RAGE) | T159+Y161 | 72 |
| Serine/threonine-protein kinase pim-2 | T195 | 72 |
| p21-activated kinase 1 (alpha) (serine/threonine-protein kinase PAK 1) (PAKa) | Pan-specific | 72 |
| Zeta-chain (TCR) associated protein-tyrosine kinase, 70 kDa | Y319 | 72 |
| Fes/Fps protein-tyrosine kinase | Y713 | 71 |
| Abelson proto-oncogene-encoded protein-tyrosine kinase | Pan-specific | 71 |
| Cyclin-dependent protein-serine kinase 1 (CDC2) | T161 | 71 |
| Protein-serine kinase C alpha (PRKCA) | Y195 | 71 |
| MAP kinase-activated protein kinase 3 | Y76 | 70 |
| Ephrin type-A receptor 2 protein-tyrosine kinase | Y588 | 69 |
| Protein kinase C-related protein-serine kinase 1 (PRKCL1, PRK1) | Pan-specific | 69 |
| Kinase homologous to SPS1/STE20 (MAP kinase kinase kinase protein-serine kinase 5 (MEKKK5) | Y31 | 69 |
| Heat shock protein HSP 90-beta (HSP90B) | Y484 | 68 |
| MAP/microtubule affinity-regulating protein-serine kinase 3 | T507 | 68 |
| Serine/threonine-protein kinase D2 (PKD2) | S197+S198 | 68 |
| Cell division cycle 7-related protein kinase | T376 | 68 |
| Heat shock 90 kDa protein alpha/beta | Pan-specific | 66 |
| Breakpoint cluster region protein | Y177 | 66 |
| Platelet-derived growth factor receptor kinase alpha | Y762 | 65 |
| Protein-serine kinase C mu (Protein kinase D) (PRKD1, PKD1, PRKCM) | Pan-specific | 65 |
| Hepatocyte growth factor (HGF) receptor-tyrosine kinase | T1355+Y1356 | 64 |
| Cyclin-dependent protein-serine kinase 1 (CDC2) | T14 | 63 |
| BR serine/threonine-protein kinase 1 | T189 | 63 |
| Cyclin E1 (CCNE1) | Pan-specific | 63 |
| Alpha-actinin-1 | Y246 | 62 |
| Cyclin B1 (CCNB1) | Pan-specific | 61 |
| Breakpoint cluster region protein | Y644 | 61 |
| Echinoderm microtubule-associated protein-like 4 | Y226 | 60 |
| RAC-beta serine/threonine-protein kinase | Pan-specific | 60 |
| Protein-serine kinase C delta (PRKCD) | Pan-specific | 60 |
| Eukaryotic translation initiation factor 4B | S422 | 60 |
| Insulin-like growth factor 1 receptor protein-tyrosine kinase | Y1280 | -62 |
| Checkpoint protein-serine kinase 1 (CHEK1) | S280 | -64 |
| Macrophage-stimulating protein receptor alpha chain (RONa) | Pan-specific | -70 |
| Glial fibrillary acidic protein | S8 | -70 |
| Insulin-like growth factor 1 receptor protein-tyrosine kinase | Y1165/Y1166 | -71 |
| Protein-serine kinase C beta 1 (PRKCB1) | Pan-specific | -73 |
| LIM domain kinase 1 | Pan-specific | -94 |
